# Follow-up and management of valvular heart disease patients with prosthetic valve: a clinical practice guideline for Indian scenario

**DOI:** 10.1007/s12055-019-00789-z

**Published:** 2019-01-28

**Authors:** Devendra Saksena, Yugal K. Mishra, S. Muralidharan, Vivek Kanhere, Pankaj Srivastava, C. P. Srivastava, VHD India consensus committee

**Affiliations:** 1 Department of Cardiac Surgery, Bombay Hospital, Mumbai, India; 2 Manipal Hospital, New Delhi, India; 3 GKNM Hospital, Coimbatore, India; 4 Chirayu Health Centre, Bhopal, India; 5 Divine Heart & Multi-speciality Hospital, Lucknow, India; 6 Narayana Hrudayalaya, Jaipur, India

**Keywords:** Valvular heart disease, Prosthetic valves, Anti-Coagulation

## Abstract

**Purpose:**

Valvular heart disease (VHD) patients after prosthetic valve implantation are at risk of thromboembolic events. Follow-up care of patients with prosthetic valve has a paramount role in reducing the morbidity and mortality. Currently, in India, there is quintessential need to stream line the follow-up care of prosthetic valve patients. This mandates the development of a consensus guideline for the antithrombotic therapy in VHD patients post prosthetic valve implantation.

**Methods:**

A national level panel was constituted comprising 13 leading cardio care experts in India who thoroughly reviewed the up to date literature, formulated the recommendations, and developed the consensus document. Later on, extensive discussions were held on this draft and the recommendations in 8 regional meetings involving 79 additional experts from the cardio care in India, to arrive at a consensus. The final consensus document is developed relying on the available evidence and/or majority consensus from all the meetings.

**Results:**

The panel recommended vitamin K antagonist (VKA) therapy with individualized target international normalized ratio (INR) in VHD patients after prosthetic valve implantation. The panel opined that management of prosthetic valve complications should be personalized on the basis of type of complications. In addition, the panel recommends to distinguish individuals with various co-morbidities and attend them appropriately.

**Conclusions:**

Anticoagulant therapy with VKA seems to be an effective option post prosthetic valve implantation in VHD patients. However, the role for non-VKA oral therapy in prosthetic valve patients and the safety and efficacy of novel oral anticoagulants in patients with bioprosthetic valve need to be studied extensively.

## Executive summary

I. Anticoagulation

A. Anticoagulants in mechanical prosthesis
  A1. Vitamin K antagonists (VKA) therapy with a target international normalized ratio (INR) range of 2.0 to 3.0 is recommended in patients with mechanical aortic valve replacement (AVR) without risk factors (grade A, EL 2).
  A2. VKA therapy with a target INR range of 2.5 to 3.5 is recommended in patients with mechanical AVR with risk factors—atrial fibrillation (AF), previous thromboembolism, left ventricular (LV) dysfunction, or hypercoagulable conditions (grade A, EL 2).
  A3. In mechanical mitral valve replacement (MVR), VKA therapy with a target INR range of 2.5 to 3.5 is recommended in patients with and without additional thromboembolic risk factors (grade A, EL 2).
  A4. With mechanical valves in both the aortic and mitral position, a target INR range of 2.5 to 3.5 is recommended (grade A, EL 2).
  A5. VKA therapy in combination with antiplatelet therapy (aspirin 75 to 150 mg) is recommended for long-term management with mechanical valve prosthesis (grade A, EL 1).
  A6. If required warfarin dose is > 10 mg, it is recommended to change to acenocoumarol; add aspirin if the patient is not already on aspirin (grade C, EL 4)
B. Anticoagulants in bioprosthesis
  B1. VKA therapy is recommended in bioprosthetic AVR and MVR, with a target INR range of 2.0 to 3.0 over no VKA therapy for 3 months (grade B, EL 3).
  B2. In patients with dual bioprosthetic valve replacement (aortic, mitral), VKA therapy is recommended for 3 months with a target INR range of 2.0 to 3.0 (grade B, EL 3).
  B3. After bioprosthetic AVR or MVR, long-term aspirin at a dose of 75 to 150 mg/day is recommended after withdrawal of VKA therapy (grade A, EL 2)
C. Anticoagulants in transcatheter aortic valve implantation
  C1. Antiplatelet agents either alone or in combination with anticoagulants are recommended after transcatheter aortic valve implantation (TAVI) (antiplatelet agents long term) (grade A, EL 2)
D. Bridging anticoagulants
  D1. It is not recommended to interrupt VKA therapy during minor dental procedures (cleaning), dermatological procedures and cataract surgery due to minimal bleeding (grade A, EL 2).
  D2. In patients with low thrombotic risk (bileaflet AVR without AF and no other risk factors for stroke), it is recommended to interrupt VKA therapy without bridging (grade B, EL 3).
  D3. Bridging anticoagulation is recommended in patients with high risk of thromboembolism during temporary interruption of VKAs (grade B, EL 3).
  D4. When interruption of VKA therapy is required, it is recommended to stop for 2–4 days before the procedure. After stopping VKA therapy, INR should be checked after 2 days and maintained < 2. The VKA therapy should be restarted 12–24 h after surgery (grade A, EL 2).
  D5. The reversal of VKA therapy during emergency surgeries can be achieved by administration of fresh frozen plasma (FFP). FFP requirement depends on the PT/INR value, liver function tests, and body weight of the patient (grade A, EL 2).
  D6. Interruption of VKA therapy with bridging anticoagulants is recommended for not more than 2 days in case of elective major surgeries (grade A, EL 3)
E. Cardiac catheterization in patients with prosthetic valves
  E1. Patients with low bleeding risk and undergoing coronary angiography with the radial approach may not require modification in VKA; however, in patients at high risk, bridging therapy is recommended (grade A, EL 1).
  E2. VKA therapy may be reduced or withheld and bridging is considered for patients with the femoral approach in the electrophysiological procedure (grade A, EL 2).
  E3. In patients who are at low risk of thromboembolic events, undergoing pulmonary vein isolation (PVI), VKA dose should be adjusted to maintain INR < 2 and regular dose to be commenced after implantation (grade A, EL 2).
  E4. In patients who are at very low risk of thromboembolic events undergoing cardiac implantable electronic devices, change in VKA therapy is not required; however, the dose should be titrated to achieve target INR < 2 (grade A, EL 2)

II. Initiation, monitoring and factors affecting anticoagulation

A. Point-of-care INR testing

- A1. The practice of patient self-management (PSM) of anticoagulation with patient self-monitoring is recommended (grade B, EL 1).
- A2. Use of point-of-care (POC) devices can be alternated with the conventional laboratory testing to reduce hospital visits and the cost of the treatment (grade B, EL 1).

B. Loading dose, dose adjustments, and frequency of INR monitoring

- B1. The initial recommended dosages of VKAs are warfarin, 5 mg and acenocoumarol, 2 mg. However, individualization of VKA dose depending on age, bleeding risk, medication compliance history, and anticipated drug interactions is also recommended (grade A, EL 2).
- B2. After baseline INR is determined, the next INR can be obtained after the patient has received two or three doses. Then after, the frequency of INR monitoring should be decided according to the value of INR (grade C, EL 4).
- B3. INR checking should be as follows: first, at discharge; second, 48 h; third, after 1 week of discharge; then after the frequency should be decided depending on INR value at first week (grade C, EL 4) (PT/INR reports 48 h after discharge as patient will be in the loading phase at the time of discharge. The fluctuation of INR for a week before the revisit after discharge not desirable).
- B4. In patients with stable INR for 3 months with no dose adjustment, it is recommended to consider INR monitoring in every 12 weeks (grade A, EL 1). However, in the case of subtherapeutic or supratherapeutic INR, the frequency of monitoring should be increased until it stabilizes again. It should be monitored 1–2 monthly in high thrombotic risk patients (grade C, EL 4).
- B5. In most asymptomatic fluctuations, the dose adjustment should be done by calculating the weekly or monthly dose of VKAs (grade C, EL 4).
- B6. For patients taking VKAs with previously stable therapeutic INRs who present with a single out-of-range INR of ≤ 0.5 below or above therapeutic INR, it is recommended continuing the current dose and testing the INR within 1 to 2 weeks with no routinely administering bridging with heparin (grade A, EL 2).
- B7. If INR falls below the target in high-risk patients, an increase in VKA dose or unfractionated heparin (UFH) may be considered (grade C, EL 4)

C. Factors affecting VKA therapy
  Pharmacogenetics

- C1. If the patient did not achieve target INR in spite of high dose of warfarin, pharmacogenetics testing may be considered, provided that it is easily available and cost-effective (grade C, EL 4).

Drug-drug interaction

- C2. It is recommended to avoid drugs that inhibit or enhance the activity of cytochrome P450 during the VKA therapy (grade A, EL 1).
- C3. It is recommended to educate patients on drug interaction with over the counter drugs and antibiotics, and need for frequent monitoring INR when they are used (grade B, EL 3).
- C4. Amiodarone, if needed, should be used with caution along with VKA therapy (grade A, EL 1).
- C5. Nonsteroidal anti-inflammatory drugs (NSAIDs) should not be used with VKAs, if at all NSAIDs are needed, paracetamol may be considered with close supervision (grade A, EL 1).

Drug-food interaction

- C6. Patients are advised to maintain constant vitamin K composition in their diet to avoid fluctuation of VKA therapy (grade C, EL 4).
- C7. Patient in special situations such as fasting, VKA therapy should be carefully monitored (grade B, EL 2).

D. Anticoagulation in special patient populations
  Pregnancy

- D1. Women with valve replacement should be advised to plan their pregnancy and inform the surgeon if the period is missed (grade C, EL 4).
- D2. Women of childbearing age should be warned about the teratogenic and harmful effects of VKAs, especially in early pregnancy (grade C, EL 4).

Before 36 weeks of gestation

- D3. Oral VKA therapy is recommended throughout pregnancy in patients with daily warfarin dose requirement of ≤ 5 mg (or equivalent acenocoumarol dose) with target INR of 3 (grade B, EL 3).
- D4. Subcutaneous UFH with activated partial thromboplastin time (aPTT) monitoring should be considered if warfarin dose is > 5 mg (also for equivalent acenocoumarol dose) (grade B, EL 3).
- D5. Low dose (75–150 mg) aspirin is recommended in second and third trimester (only in high-risk patients or all patients or in stable patients to reduce INR by 0.5) (grade C, EL 4).

At 36 weeks of gestation

- D6. If the patient is hospitalized, VKA may be substituted with UFH. If not, VKA therapy should be discontinued prior to admission for delivery (grade C, EL 4).
- D7. When hemostasis is adequate, VKA therapy should be restarted on day 1 at maintenance dose along with heparin (grade C, EL 4).

Elderly population

- D8. Frequent renal tests and observation for adverse effects with concomitant medications are recommended in elderly patients considering them as high-risk patients for developing hemorrhagic complications (grade B, EL 3).

Cancer

- D9. In patients with chemotherapy, VKA therapy should be closely monitored (grade A, EL 2).

Renal impairment

- D10. Renal impairment patients should be closely monitored (grade B, EL 3).

III. Management of prosthetic valve complications

A. Thromboembolic events

- A1. Transthoracic echocardiogram (TTE) is recommended for the diagnosis of thromboembolic events (grade A, EL 2).
- A2. Treatment with tissue plasminogen activator (tPA) and heparin is recommended in patients with stroke; other vascular occlusions should be managed by surgery (grade C, EL 4).
- A3. In anticoagulant patients with thromboembolic events, daily aspirin (75 to 81 mg) is recommended with an increase in the target INR range (mechanical AVR: 2.5–3, mechanical MVR: 3–4) (grade C, EL 4).
- A4. In patients with bioprosthetic valve, who are only on aspirin, addition of VKAs can be considered (grade B, EL 3).
- A5. Measures to increase patient compliance (patient education) are recommended in all patients with thromboembolic events (grade C, EL 4).

B. Thrombosis of prosthetic valves

- B1. TTE is recommended in patients with suspected prosthetic valve thrombosis to assess hemodynamic severity and resolution of valve dysfunction (grade B, EL 3). Cinefluoroscopy can be considered as an additional tool for diagnosis of prosthetic valve thrombosis (PVT) (grade C, EL 4).
- B2. Transesophageal echocardiogram (TEE) is recommended to assess thrombus size and valve motion (grade B, EL 3).
- B3. UFH is recommended in a very small and non-obstructive thrombus burden of left-sided PVT. If not treated, fibrinolytics are recommended (grade B, EL 3).
- B4. In patients with left-sided PVT, with thrombus burden, ≤ 0.8 cm^2^ fibrinolytics are recommended over surgery (grade B, EL 3).
- B5. Emergency surgery is recommended in case of left-sided PVT with a mobile or large thrombus > 0.8cm^2^. Fibrinolytics can be considered in patients with contraindications to surgery (grade B, EL 3).
- B6. The right-sided thrombosis can be treated with fibrinolytics if no contraindications to fibrinolytics are present. If fibrinolytic therapy is successful, intravenous (IV) UFH is recommended until the patient achieves an INR of 3.0 to 4.0 for aortic prosthetic and 3.5 to 4.5 for mitral prosthetic valves (grade B, EL 3)

C. Bleeding complications

- C1. In absence of active bleeding and INR in the range of 4.5–10.0, it is recommended withholding VKA with serial INR determination, and resuming when INR is therapeutic (grade B, EL 3).
- C2. In absence of active bleeding, and INR > 10, it is recommended withholding VKA and administering FFP and should be hospitalized (grade C, EL 4).
- C3. For patients with VKA-associated active bleeding, withhold VKA, and administer FFP. It is also recommended to administer vitamin K1 as slow IV infusion if uncontrolled (grade A, EL 2)

D. Prosthetic valve endocarditis (PVE)

Diagnosis

- D1. TTE is recommended in suspected infective endocarditis (IE), in the case of negative TTE in suspected PVE, TEE is recommended. If initial examinations are negative, repetition of TTE/TEE is recommended within 7–10 days in patients with high suspicion of IE. Modified Duke Criteria should be used in evaluating a patient with suspected IE (grade B, EL 3).

Prophylaxis

- D2. Antibiotic prophylaxis is recommended for certain dental procedures like gingival or periapical (root) procedures with perforation of the mucosa, and also for infected gastrointestinal and urogenital tract procedures (grade C, EL 4).

Antithrombotic

- D3. It is recommended to discontinue VKA, in patients on VKA for a prosthetic valve who develop IE, until it is clear that invasive procedures will not be required and the patient has stabilized without signs of central nervous system (CNS) involvement. When the patient is deemed stable without contraindications or neurologic complications, reinstitution of VKA therapy is recommended (grade B, EL 3)

IV. Follow-up evaluations and management of concomitant cardiac disease

A. Heart failure

- A1. Antithrombotics are recommended in valve replacement patients with stage A heart failure (HF) (grade A, EL 1). Angiotensin-converting enzyme (ACE) inhibitors and β blockers could be beneficial if added in older patients (grade B, EL 3).
- A2. ACE inhibitors and β blockers along with antithrombotics are recommended in patients with stage B, C HF (grade A, EL 1). Moreover, spironolactone and digoxin can be added if congestive failure supervenes despite regular medications (grade A, EL 2).
- A3. In patients with stage D HF, other interventions including cardiac transplant are recommended (grade D, EL 4)

B. Coronary artery disease

- B1. Bare metal stents are recommended over drug-eluting stents in patients with valve replacement to lower bleeding risk (grade D, EL 4)

C. Atrial fibrillation

- C1. Prophylactic amiodarone is recommended as a routine therapy for high-risk patients undergoing cardiac surgery in absence of a contraindication (grade A, EL 1).
- C2. Amiodarone at a dose of 100–200 mg daily for 3 months with dosage monitoring along with β blockers is recommended for transient perioperative AF (grade A, EL 2)

D. Follow up cardiac evaluation

- D1. First postoperative visit to a cardiac specialist should be within 1 month of discharge (grade A, evidence level 4).
- D2. The timings of echocardiographic examination: first, at pre-discharge; second, at 1 month; then yearly; and should be done at any time when symptoms occur (grade C, EL 4).
- D3. The patient should be followed up by TTE and/or TEE in case of clinical symptoms or signs of prosthetic valve dysfunction (grade C, EL 4).
- D4. Echocardiographic testing is recommended for (a) unexplained cardiac symptoms, (b) annually in all patients with congestive heart failure (CHF). Moreover, echocardiography is indicated whenever there is an episode of thromboembolism (grade C, EL 4).
- D5. Mechanical heart valve patients should undergo annual follow up in presence of a change in clinical status (grade C, EL 4)

E. CT and MRI scan-post valve implantation

- E1. Magnetic resonance imaging (MRI) examination (3 T or less) is safe in patients with a prosthetic heart valve or annuloplasty ring or sternal wire (grade C, EL 3).
- E2. MRI examination for patients with risk of endocarditis and valve dehiscence should be decided in consultation with a radiologist (grade D, EL 4).

## Introduction

Valvular heart disease (VHD) is one of the common causes of cardiac morbidity and mortality [1]. The burden of VHD is growing worldwide due to the high incidence of rheumatic heart disease (RHD), especially in developing countries, and due to the increase in degenerative etiologies in industrialized nations [2, 3]. Prevalence of VHD in industrialized countries is estimated at 2.5% [2]. Data on the burden of RHD in India comes from hospital data (20–50%), population-based studies (2.2–1.6%), and school surveys (0.67–4.54%) [4]. The pattern of valve involvement is mitral (54.4%), aortic (17.8%), both mitral and aortic (18.0%), tricuspid (9.7%), or pulmonary (0.04%). Overall, RHD contributes 63.4% to the prevalence of VHD [5]. This pattern of VHD in India is in contrast to the developed countries, where the most frequently involved valve type is aortic with degenerative etiology [6, 7]. Surgical valve repair or replacement using either a mechanical or bioprosthetic valve is a common solution practiced globally to manage or treat VHD.

The worldwide annual rate of valve replacement is projected around 275,000 to 370,000, of which 55% are mechanical heart valves and 45% are bioprosthesis heart valves [8, 9]. In India, this number is estimated to be in excess of 10,000 [10]. Globally, the prosthetic valve implantations are increasing at a rate of 5–7% per year with bioprosthesis valves gaining favor over mechanical heart valves; 8–11% versus 3–5% per year respectively [11]. This increase may be attributed to increasing rate of valve replacement surgery in the elderly, in whom bioprosthetic is preferred, and to technological advances in bioprosthesis compared to mechanical device development [12–14].

Globally, valve repair rate for isolated mitral valve disease from 2000 to 2007 has increased from 51 to 69%. However, this rate is still much lower than the 90% or higher rates achieved by some institutions [15–17]. Additionally, the repair rate among the elderly has remained much lower than their younger counterparts [18]. Valve repair surgery has some short-term advantages such as minimal morbidity and mortality, better survival, superior preservation of left ventricular (LV) function, and less valve-related complications over valve replacement surgery [19]. Furthermore, evidence indicates that along with short-term benefit, patients with valve repair also have an extra advantage toward the long-term survival compared to valve replacement patients [18, 20]. Management of patients with valve repair necessitates antithrombotic therapy for the first 3 months following surgery [21], which is a high-risk period for thromboembolism. Anticoagulation therapy has no extra advantage over antiplatelet therapy in valve repair patients, and anticoagulation therapy may lead to major bleeding complications [22]. Nonetheless, the efficacy of anticoagulants in patients with valve repair needs to be confirmed in randomized controlled trials (RCTs).

### Rationale

An expert analysis of evidence related to drugs, devices, and procedures for diagnosis, management, and prevention of disease, direct toward improvement in the quality of care. Evaluation of data on the benefits and risks of these therapies can optimize patient outcomes and favorably affect costs by focusing resources on the most effective strategies.

Patients with prosthetic valves require lifelong follow-up and anticoagulant therapy. Failure in attaining appropriate follow-up care can lead to life-threatening complications. In order to alleviate morbidity and mortality in these patients, the Indian Association of Cardiovascular-Thoracic Surgeons (IACTS) has set forth to develop guidelines for follow-up care of patients with prosthetic valves, specifically gathering the evidence and formulating the recommendations with Indian perspective.

### Methodology

The current Good Clinical Practice Recommendations (GCPR) from the IACTS for the follow-up and management of VHD patients with a prosthetic valve in India is developed by an “expert panel” of cardiologists and cardiothoracic surgeons across the country with vast experience in managing prosthetic valve patients.

A national panel with 13 members reviewed the literature and collected the evidence. Literature search was carried out electronically in the medical search engine “PubMed” and Google Scholar for relevant reports published between 1990 and October 2017. The main search strategy included the following keywords: valvular heart disease, prosthetic valves, anticoagulation with no limitation of time, and India (to identify specific evidence). Also, a manual search was made from key non-indexed journals. Abstracts written only in English were included. Evidence from RCTs and non-RCTs, retrospective and uncontrolled studies, reviews and meta-analyses were considered for framing the GCPR. When evidence was scant for specific areas, existing recommendations from national and international guidelines for the management of prosthetic valve patients were carefully analyzed.

Expert panel members reviewed the draft guideline along with the proposed GCPR, and a series of discussions were made to arrive at a consensus on each GCPR. Later on, extensive discussions were held on this draft and the recommendations in 8 regional meetings involving 79 additional experts to arrive at a consensus. The final consensus document is developed relying on the available evidence and/or majority consensus from all the meetings. When evidence is weak or does not exist for certain areas, the consensus opinion of the expert panel has been relied upon. Recommendations were graded as per the American Association of Clinical Endocrinologists (AACE) guidelines (Table 1) [23].

**Table 1:** Grading of recommendations

Strength of recommendation	
A	Strong recommendation
B	Intermediate recommendation
C	Weak recommendation
D	No evidence-based recommendation
Scale of scientific support (evidence level)	
1	At least one randomized controlled trial (RCT) or meta-analysis of RCTs
2	At least one non-randomized/non-controlled, prospective epidemiological study
3	Cross-sectional or observational or surveillance or pilot study
4	Existing guideline or consensus expert opinion on extensive patient experience or review

## Anticoagulation

Prosthetic valve implantation requires postoperative prophylactic anticoagulation to preclude thrombotic events, which are the common cause of morbidity and mortality after surgery in patients with VHD. A paramount risk of thromboembolic events is observed during the first 3 months after surgery for both mechanical and bioprosthetic devices. Nevertheless, mechanical valves exhibit lifetime thrombotic risk. Atrial fibrillation (AF), which is common in VHD, necessitates lifelong anticoagulation in the majority of patients; especially if it involves mitral valve. Moreover, patients on anticoagulants are always at risk of thrombosis and bleeding complications if the target international normalized ratio (INR) levels are not maintained. Restrictions on certain physical activities that are advised subsequently after surgery to reduce chances of bleeding accidents compromise lifestyle of the young patients. These considerations emphasize the importance of addressing proper anticoagulation techniques to minimize postoperative thrombotic complications while maintaining acceptable levels of risk related to bleeding.

### Anticoagulants in mechanical prosthesis

Apart from the inherent thrombogenic characteristics of the intravascular prosthetic material, mechanical valves impose abnormal flow conditions within their components, with zones of low flow and areas of high-shear stress. These can cause platelet activation and lead to valve thrombosis and thromboembolic events.

#### The studies comparing lower and higher INR targets

The difference in outcomes between lower and higher INR targets in patients with valve replacement is controversial. The AREVA trial has failed to demonstrate or exclude a beneficial or detrimental effect of higher versus lower INR (3.0 to 4.5 vs. 2.0 to 3.0), achieved with Vitamin K antagonist (VKA), on thrombosis or hemorrhage in patients who predominantly had aortic valves [24]. LOWERing the INtensity of oral anticoaGulant Therapy in patients with bileaflet mechanical aortic valve replacement (AVR) (LOWERING-IT) trial compared INR target of 1.5 to 2.5 to the conventional 2.0 to 3.0 in low-risk bileaflet mechanical aortic valve patients who are on daily oral coumadin therapy without aspirin. The trial proposed that low INR target is safe and feasible for low-risk patients. However, the study had a low quality of evidence owing to imprecision due to only four thrombotic events [25]. The German Experience with Low-Intensity Anticoagulation (GELIA) trial has shown the difference in the clinical outcomes by INR target for aortic versus mitral prostheses following coumadin anticoagulation. Thromboembolism following AVR was significantly lower than after mitral valve replacement (MVR). In the end, the trial support re-examination of the intensity of anticoagulation in patients with the St. Jude Medical valve. However, there was a trend toward fewer thromboembolic events in AVR patients with a higher INR in the trial [26]. A prospective study after evaluating 4202 patients with a mechanical valve, AF, or myocardial infarction (MI) reports that the optimal anticoagulation for patients with mechanical heart valve prostheses was achieved with an INR of 2.5 to 2.9, an INR of 3.0 to 3.4 for patients with AF, and an INR of 3.5 to 3.9 for patients after MI [27].

Cannegieter and associates investigated the optimal intensity of oral anticoagulation therapy (OAT) in 1608 patients with mechanical heart valves. The optimal intensity of anticoagulation achieved with VKA, defined as the INR level with the lowest incidence of both bleeding and thromboembolism, was in the range of 2.5 to 4.9. Furthermore, a target range of 3.0 to 3.9 was found better than a target of 2.0 to 2.9 in a subgroup of mitral valve patients. However, the study was underpowered for subgroup analysis [28]. Another study by Pengo V et al. reports fewer bleeding events at target INR 3 compared to INR 4 achieved with VKA. The thrombotic events were similar in both the groups [29]. Furthermore, American college of chest physicians (ACCP) suggests that addition of antiplatelet agent (APA) to VKAs is associated with a significant reduction in mortality and thromboembolic outcomes with a relatively small increase in the risk of major hemorrhage [30].

#### Novel mechanical valves with proposed lower INR targets

A new generation mechanical valve, On-X mechanical valve (Medical Carbon Research Institute, Austin, TX, USA), has been shown to exhibit low thrombogenicity [31]. In the Prospective Randomized On-X Anticoagulation Clinical Trial (PROACT), AVR patients were randomized to receive warfarin at either low dose (INR 1.5–2.0) or a standard dose (INR 2.0–3.0) for 3 months following surgery. Daily aspirin (81 mg) was given to all patients. Mean INR was 1.89 with low dose warfarin and 2.50 with standard treatment (*p* < 0.0001). Major and minor bleeding rates were significantly lower in low-dose group, and there was no significant difference between the two groups in terms of stroke, transient ischemic attack (TIA), or total neurological events and all-cause mortality [32]. A recent case report advocate that lower level of anticoagulation may be suitable in patients with On-X valves and proposes that On-X valve may be helpful when therapeutic levels of anticoagulation cannot be attained due to the increased risk of bleeding [33].

##### TTK Chitra prosthetic heart valve (CHV)

High prevalence of rheumatic valvular disease and the high cost of imports bolstered the need for the development of an Indian valve. As a result, the TTK Chitra prosthetic heart valve (CHV) was developed, which is the first heart valve to be manufactured in India that has been used since 1992 [34]. This tilting disc valve has an integrally machined cobalt alloy cage, an ultra-high molecular weight polyethylene disc, and a polyester suture ring. Sankarkumar R. et al. evaluated the outcomes of CHV in 285 survivors with isolated MVR or AVR for a total of 1212 patient-years (pt-yr) (AVR, 445 pt-yr; MVR, 767 pt-yr). There was no incidence of a paravalvular leak or structural dysfunction of the valve. The study reported bleeding events (AVR 0.9 per pt-yr, MVR 0.4 per pt-yr) and thromboembolism (AVR 1.6% per pt-yr, MVR, 2.4% per pt-yr) with an actuarial survival of 82.4 ± 4.0% for AVR and 65.2 ± 5.0% for MVR at 7 years [34]. Similarly, Muralidharan S et al. reported 10-year outcomes of patients (*n* = 65) who underwent valve replacement with CHV [MVR, 58.5%; AVR, 29.3%; dual valve replacement (DVR), 12.3%]. All patients maintained with anticoagulation acenocoumarol, warfarin, or phenindione at INR of 2.5–3.0. The study reported the incidence of valve thrombosis (4.6%), prosthetic valve endocarditis (PVE) (4.6%), MI (2.3%), and the low cardiac output secondary to severe LV dysfunction (2.3%) with a long-term mortality rate of 20.9%. CHV has acceptable thromboembolic levels with low cost and has been the valve of choice in a large number of centers dealing with RHD in the lower socioeconomic strata of society [35].

#### Replacement of VKA therapy by antiplatelet agents

Current evidence does not support the replacement of VKA therapy with APA for either mechanical aortic or mitral valve prostheses. Earlier studies in the pediatric population have demonstrated an unacceptable risk of thromboembolism when treating with APA alone [36, 37]. In Clopidogrel and aspirin in the prevention of thromboembolic complications after mechanical aortic valve replacement (CAPTA) trial, patients with mechanical AVR were randomized to OAT versus APA. The trial was stopped after valve thrombosis events were reported in one patient [38]. Indirect evidence from this trial in AF provides strong support for the effectiveness of VKA over APA in patients with mechanical valves. ACCP demonstrates an increased risk of major hemorrhage for those targeted for a higher INR (INR 3.0–9.0). Moreover, there exists no evidence which state that high INR for mechanical AVR results in fewer thrombotic events. Furthermore, no evidence demonstrates that higher INR targets have an additional benefit over harm in patients with risk factors [30].

#### Indian evidence

In a prospective observational study, Dhanya PS et al. evaluated the pattern of OAT use, achievement of target INR, mean dose of warfarin and acenocoumarol, and the anticoagulation-related adverse events (AEs). The therapeutic range (TR) of INR was defined as 2.0–3.0 in the AVR patients and 2.5–3.5 in the MVR and DVR patients. The study included 70.9% of MVR patients. Warfarin was used in 58.2% patients (mean INR, 2.52 ± 0.81; mean dose of 3.68 ± 1.71 mg) with time in TR 44.8%. Acenocoumarol was used in 44.8% patients (mean INR, 2.76 ± 0.51; mean dose of 3.13 ± 1.23 mg) with time in TR 44.2%. Thromboembolic complications were reported in 3.6% patients; only when the mean INR < 1.6. The authors conclude that target therapeutic range (TTR) can be broadened to 1.6–4.0; however, they identified the need of RCTs to fix a lower TR in Indian population [39].

John S et al. have reported their 25 years of experience with Starr Edward’s ball valves in patients with combined MVR and AVR. The target prothrombin time (PT) was maintained at 1.5 times the control value with a low-intensity anticoagulant. A thromboembolic event of 1.05/100 pt-yr and anticoagulant-related bleeding of 0.12/100 pt-yr was reported [40].

A retrospective study evaluated the complications and outcomes in 88 patients after replacement with Starr-Edwards, St. Jude, and Medtronic-Hall valves in mitral, aortic, or both positions. The study reported that St. Jude valve in mitral position had the highest thromboembolic risk of 12.5/100 pt-yr among the single valve replacement group, and all prostheses had a high thrombotic risk in the DVR group: St. Jude 22.2, Medtronic-Hall 12.5, and Starr-Edwards 8.6 per 100 pt-yr [41]. Panda BR et al. followed up 382 patients with mechanical valves in both positions for 15 years. The authors conclude that protection against thromboembolism and anticoagulation-related hemorrhage can be boosted with a strict adherence to optimal anticoagulation level, and it also helps to provide the patient with a good quality of life (QoL) [42].

Moreover, an article published in Journal of the Association of Physicians of India (JAPI) concluded that although warfarin is a choice of anticoagulant in the United States (US), acenocoumarol is being widely used in Indian patients with valve replacement. Especially in North India, acenocoumarol is widely used in place of warfarin [43].

#### Evidence from other Asian countries

A randomized trial compared the outcomes of moderate (INR, 2.65) versus high (INR, 9) intensity anticoagulation in 258 mechanical prosthetic heart valve patients. Study reported more bleeding episodes in high-intensity group (12.1/100 patient-years) compared to moderate-intensity group (6.2/100 patient-years) (*p* < 0.002). Authors conclude that both groups have equivalent protection, but the moderate anticoagulation group had a significantly lower bleeding risk [44]. Akhtar RP et al. prospectively evaluated 507 prosthetic valve patients on warfarin anticoagulation in Pakistan. A total of 1.13% per patient year of thromboembolism and 2.04% per patient year of bleeding events were observed with mean INR 2.6 ± 0.59. The study included 52.9%, 18.9%, and 15.0% patients with MVR, DVR, and AVR respectively [45].

A prospective study from China included 45%, 27%, and 28% of patients with MVR, AVR, and DVR. The target INR range was 1.4 to 1.9 for patients with AVR and 1.5–2.0 for those with MVR and DVR. The mean INR reported was 1.68 ± 0.38. The study reported a linearized rate of 5.83% per pt-yr for bleeding and 0.26% per pt-yr for thromboembolic events after CarboMedics mechanical valve implantation [46]. In a prospective study by Sun X et al., a total of 230, 318, 189, and 5 patients were enrolled with AVR, MVR, DVR, and tricuspid valve replacement (TVR) respectively. The target INR of 2.0–2.5 was achieved with OAT. There were 1.59% per pt-yr of hemorrhagic events, 0.34% per pt-yr of thromboembolic, and 0.19% per pt-yr of thrombosis after prosthesis following St Jude Medical valve implantation for Chinese patients [47]. In a study by Matsuyama K et al., a total of 214 patients were followed retrospectively after mechanical MVR (mean duration of follow-up, 4.8 years; total duration of follow-up, 1027 pt-yrs) on OAT with or without aspirin, ticlopidine, or dipyridamole. The target INR was between 1.5 and 2.5. Thromboembolism was observed in 0.8% per pt-yr and major bleeding in 0.5% per pt-yr) [48].

A study conducted in Japan concluded that the optimal INR of 2.5–3.5 for patients on OAT recommended by the American Heart Association (AHA) might be too high in Japanese patients, and INR < 2.5 may be safe to prevent hemorrhagic complications [49].

The target INR ranges recommended by American College of Cardiology (ACC)/AHA and ACCP for mechanical valve replacement are comparable. The current ACCP recommendations relate mostly to bileaflet and other new generation valves. ACC/AHA recommends a target INR of 3.0 for patients with older generation mechanical aortic valves (cage and ball), while ACCP suggests the target INR 2.0–3.0. They also noted high rate of major hemorrhage with an INR that is even somewhat lower, 3.0–4.5. However, the problem is self-limited, because few such valves are being inserted [50]. Both the guidelines [30, 51] recommend aspirin in addition to VKA in mechanical valve patients who do not have contraindications to aspirin (i.e., bleeding or aspirin intolerance); however, the dosage slightly varies (ACC/AHA: 75–100 mg/day, ACCP: 50–100 mg/day). Furthermore, ACC/AHA emphasizes the use of aspirin in patients with a history of embolus while on VKA therapy with a therapeutic INR, those with the known vascular disease, and those who are known to be particularly hypercoagulable. For patients with mechanical aortic or mitral valves, ACCP recommends VKA over antiplatelet agents. The target INR for mechanical valve patients according to above guidelines is summarized in Table 2.

**Table 2:** The target INR values for prosthetic valve patients on VKA

Type of valve	Target INR			
	Aortic position		Mitral position	
	Uncomplicated with no risk factors^a^	With risk factors	Uncomplicated with no risk factors^a^	With risk factors
Mechanical valve^b^				
Bileaflet	2.0–3.0	2.5–3.5	2.5–3.5	2.5–3.5
Tilting disc	2.0–3.0	2.5–3.5	2.5–3.5	2.5–3.5
Caged ball	2.5–3.5	2.5–3.5	2.5–3.5	2.5–3.5
Caged disc valve	2.5–3.5	2.5–3.5	2.5–3.5	2.5–3.5
Bioprosthetic valve^c^				
Xenograft	2.0–3.0	2.0–3.0	2.0–3.0	2.0–3.0

^a^AF, previous thromboembolism, LV dysfunction, or hypercoagulable conditions

^b^VKA plus aspirin, class I indication by ACC (75–100 mg OD), ACCP (50–100 mg OD) recommends aspirin only in high-risk patients

^c^VKA for 3 months in all except uncomplicated aortic valve replacement where ACCP recommends aspirin over VKA and it should be continued if there is no other indication of anticoagulation. Postoperative aspirin (80–100 mg OD) is recommended for all by ACC, while ACCP recommends aspirin 3 months after replacement

### Indian recommendations

- VKA therapy with a target INR range of 2.0 to 3.0 is recommended in patients with mechanical aortic valve replacement without risk factors (grade A, EL 2).
- VKA therapy with a target INR range of 2.5 to 3.5 is recommended in patients with mechanical aortic valve replacement with risk factors—AF, previous thromboembolism, LV dysfunction, or hypercoagulable conditions (grade A, EL 2).
- In mechanical mitral valve replacement, VKA therapy with a target INR range of 2.5 to 3.5 is recommended in patients with and without additional thromboembolic risk factors (grade A, EL 2).
- With mechanical valves in both the aortic and mitral position, a target INR range of 2.5 to 3.5 is recommended (grade A, EL 2).
- VKA therapy in combination with antiplatelet therapy (aspirin 75–150 mg) is recommended for long-term management with mechanical valve prosthesis (Grade A, EL 1).
- If required warfarin dose is > 10 mg, it is recommended to change to acenocoumarol; and add aspirin if the patient is not already on aspirin (grade C, EL 4).

### Anticoagulants in bioprosthesis

Thromboembolic events with bioprosthetic valves have been reported to range from 0.2 to 3.3% per year. The risk is higher in the MVR compared to AVR [18, 52, 53]. The lack of complete endothelialization of suture zone in the early postoperative period may contribute to the thrombogenicity of bioprosthesis [53].

The optimal antithrombotic regimen and its duration after placement of a bioprosthetic device are less clear. Studies have demonstrated that bioprosthetic devices have an increased risk for thromboembolic events during the first three months after the procedure which is less than that associated with mechanical valves [54, 55].

Evidence regarding the use of VKAs and aspirin in patients with bioprosthesis valve is contradicting. A prospective study (ACTION Registry) was conducted in 47 medical centers in Europe, Canada, and India with 1118 patients who underwent AVR alone or combined with coronary artery bypass graft (CABG); 500 patients in the study received a VKA and 618 received aspirin. Major bleeding or thromboembolism occurred in 6% patients treated with VKA and 2.8% patients treated with aspirin (*p* = 0.003). The study suggested that, particularly after concomitant CABG surgery, recipients of bioprosthetic AVR should receive prophylactic aspirin instead of VKA [56]. In a cohort study by Sundt TM et al., patients with and without anticoagulation were evaluated for neurologic events within 90 days of bioprosthetic AVR. The postoperative cerebrovascular accident occurred in 2.4% patients on anticoagulation and 1.9% without anticoagulation. The postoperative cerebrovascular accident was found unrelated to warfarin use. The authors concluded that early anticoagulation with warfarin after bioprosthetic AVR did not appear to protect against neurologic events [57]. A Danish nationwide study investigated the association of warfarin treatment with the risk of thromboembolic complications, bleeding incidents, and cardiovascular deaths after bioprosthetic AVR and found that discontinuation of warfarin treatment within 6 months after bioprosthetic AVR was associated with increased cardiovascular death [58]. In a prospective study, Al-Atassi T et al. have demonstrated that warfarin plus aspirin and aspirin only have equivalent effects on cerebral micro-embolization and platelet function at 1 month after implantation of bioprosthetic aortic valves. They proposed only aspirin in patients who have no other indication for OAT early after implantation of bioprosthetic aortic valves [59]. In an analysis from a national database, the risks and benefits of short-term anticoagulation in patients with bioprosthesis AVR were evaluated. The study concluded that although there is a more risk of bleeding associated with aspirin plus warfarin, death and embolic events were relatively rare in the first 3 months after bioprosthetic AVR in both the groups [60].

Agarwal S et al. compared outcomes of mechanical versus bioprosthetic MVR in patients between 40 and 60 years of age. Bioprosthetic valve patients received dicoumarol only for 3 months while aspirin was continued indefinitely and the target INR was set as 2.5–3.5. The rate of valve thrombosis and bleeding was more with mechanical valve than in patients with bioprosthetic valves. The authors concluded implantation of a bioprosthetic valve in patients with this age group [61]. Abraham S et al. evaluated the outcomes of Carpentier Edwards (Porcine) bioprosthesis in 18 MVR, 14 AVR, 1 TVR, and 12 DVR patients. All patients were on OAT and APA for 3 months. The rate of thromboembolism and infective endocarditis (IE) was 2.4%. No incidence of anticoagulant-related hemorrhage was observed during the follow-up [62]. Talwar S et al. also evaluated the outcomes in Carpentier Edwards (Porcine) bioprosthesis. The target INR was 2.0–3.0. All patients received coumadin for first 6 weeks postoperatively, and indefinite aspirin 150 mg/day. There were seven episodes of thromboembolism (0.64% events/pt-yr), and one episode of hemorrhage [63]. Mandiye SS et al. studied short-term outcomes after MVR, AVR, and DVR with mechanical versus bioprosthetic valves. All patients postoperatively received dicoumarol, and aspirin 150 mg/day. The target INR was 2.0–3.0 in MVR or AVR, and 2.5–3.5 in DVR. The study concluded that mechanical valves have significantly higher complication rate than bioprosthesis valves in Indian patients [64].

Recommendations from the ACCP and ACC/AHA contradict each other about the antithrombotic therapy in the bioprosthetic valve in the postoperative period. The ACCP currently recommends VKA therapy with target INR 2.5 for first 3 months after bioprosthetic MVR. In patients with no other indication for anticoagulation (i.e., atrial dysrhythmias, history of thromboembolism, etc.), ACCP recommends aspirin (50 to 100 mg/day) over VKA therapy for the first 3 months after surgery for AVR with a bioprosthetic device. Moreover, in all patients with bioprosthesis, ACCP recommends continuation of aspirin therapy without VKA therapy beyond the initial 3-month postoperative period if the patient remains without a definitive indication for anticoagulation [30]. The ACC/AHA guidelines do not have strong recommendations supporting the use of VKA therapy in patients with bioprosthetic valves [51]. However, they suggest VKA therapy is reasonable for the first 3 months after bioprosthetic MVR and in patients with bioprosthetic AVR VKA therapy might be effective for the first 3 months after valve replacement [51]. Aspirin therapy at a dose of 75 to 100 mg/day is recommended in all bioprosthetic valve patients regardless of whether anticoagulation is employed [51]. Moreover, there are no specific recommendations offered with regard to duration of aspirin therapy in this population. The choice of the antithrombotic regimen in the setting of bioprosthetic valve replacement is largely left to the individual clinicians in ACC/AHA guidelines. The duration and intensity of aspirin treatment are also left to the individual clinician’s discretion. Several factors including institutional-specific outcomes, the likelihood of patient adherence to medication regimen, prior personal experience, regional convention, and personal preference may influence a clinician’s decision. The target INR for mechanical valve patients according to above guidelines is summarized in Table 2.

### Indian recommendations

- VKA therapy is recommended in the bioprosthetic aortic valve and mitral valve replacement, with a target INR range of 2.0 to 3.0 over no VKA therapy for 3 months (grade B, EL 3).
- In patients with dual bioprosthetic valve replacement (aortic, mitral), VKA therapy is recommended for 3 months with a target INR range of 2.0 to 3.0 (grade B, EL 3).
- After bioprosthetic aortic or mitral valve replacement, long-term aspirin at a dose of 75 to 150 mg/day is recommended after withdrawal of VKA therapy (grade A, EL 2).

### Anticoagulants in transcatheter aortic valve implantation

Transcatheter aortic valve implantation (TAVI) has become established as a treatment option for patients with symptomatic aortic stenosis (AS). In comparison with surgical AVR, TAVI offers superior quality of life with similar mortality rates among patients at very high surgical risk [65]. However, thromboembolic complications from TAVI are significant, and stroke, in particular, is a concern [66]. While the immediate procedural risk relates to valvular debris embolization, 50% of strokes develop after the first day and may relate to non-procedural events [65–67]. The incidence of cerebrovascular events after TAVI remains high for at least 60 days. This implies that the prothrombotic environment of the bioprosthesis itself may be implicated in distal thromboembolism, and therefore antiplatelet or antithrombotic treatment should play an important role in stroke prevention [68].

Various combinations of antithrombotic regimens (single-APA, dual-APA, or VKAs) have been used, but evidence-based guidance remains lacking. The benefit of TAVI with the core valve revolving system was evaluated in a prospective trial. All patients received aspirin 100 mg before the procedure, which is continued lifelong. A loading dose of clopidogrel 300 mg was administered the day before the procedure followed by 75 mg daily for 3 to 6 months. The cumulative incidences of mortality were 5.4% at 30 days, 12.2% at 6 months, and 15.0% at 1 year [69]. In a randomized multicenter PARTNER trial, TAVI was compared to surgical AVR in high-risk patients. All the patients received heparin during the procedure and dual APAs (aspirin and clopidogrel) for 6 months afterwards. The rates major stroke (*p* = 0.07) and major vascular complications were more frequent in TAVI patients at 1 year (*p* < 0.001) [65]. In a randomized trial, the clinical outcomes of standard therapy (including balloon aortic valvuloplasty) versus TAVI (balloon-expandable bovine pericardial valve) in patients with severe AS, not suitable for surgery, were compared. Adjunctive pharmacologic therapy included heparin during the procedure and dual APAs (aspirin and clopidogrel) for 6 months after the procedure. Despite the higher incidence of major strokes and major vascular events at 1 year with TAVI, it significantly reduced the rates of death from any cause, the composite endpoint of death from any cause or repeat hospitalization, and cardiac symptoms, compared with standard therapy [66].

In a prospective study, cerebral embolization during TAVI was evaluated at 3 months after the surgery. All patients received aspirin (100 mg/day), clopidogrel (75 mg/day after a loading dose of 300 mg/day) before the procedure; clopidogrel was discontinued after 6 months, while aspirin continued indefinitely. Procedural micro-embolization signals were detected in all patients. No embolization was found at 3 months follow-up [67]. The risk of cerebrovascular events at discharge, at 1 and 6 months, and at yearly was evaluated in a subgroup of patients enrolled in a randomized PARTNER trial. Clopidogrel loading dose was given 6 h before the procedure in 134 patients, whereas 34 patients were already on a steady dose of 75 mg daily for > 1 week. Clopidogrel was not given before TAVI to patients undergoing the transapical procedure and clopidogrel maintenance dose was discontinued for 5 days before TAVI in eight patients. Ninety-three percent of patients were on aspirin before TAVI. At discharge, 89%, 59%, and 33% patients were on aspirin, clopidogrel, and antithrombotic therapy (heparin or warfarin), respectively. Among patients who developed cerebrovascular events, 91% were on aspirin, 61% were on clopidogrel, and 35% were receiving antithrombotic therapy at the time of the event [68]. It is to be noted that none of these trials had measured bleeding or thromboembolic events as the primary objectives.

In a prospective cohort study, Poliacikova P et al. compared procedural and follow-up complications of TAVI patients based on the type of antithrombotic treatment used (single-APA vs. dual-APA vs. warfarin). A total of 34%, 53%, and 13% patients were on dual-APA, single-APA, and warfarin, respectively. The combined endpoint of all-cause death, acute coronary events, stroke, or bleeding was significantly worse in the dual-APA group. The occurrence of major adverse cardiac and cerebrovascular events was statistically similar in all groups. The results suggest that dual-APA did not protect patients from stroke and may expose them to higher bleeding risk [70].

There are only two case reports on initial Indian experience of TAVI [71, 72]. The recent American College of Cardiology Foundation (ACCF)/American Association for Thoracic Surgery (AATS)/Society for Cardiovascular Angiography and Interventions (SCAI)/Society of Thoracic Surgeons (STS) expert consensus document on TAVI did not make much clarity on this difficult area [73]. Rodés-Cabau et al. have summarized the recommendations for antithrombotic therapy in TAVI [74]. The overall comparison of antithrombotic recommendations is presented in Table 3.

**Table 3:** Antithrombotic recommendations in patients undergoing TAVI

Recommendations	Pre-procedural	Procedural	Post-procedural (first 30 days)
PARTNER Trial [65, 66]	Aspirin 80 mg, Clopidogrel 300 mg	UFH, Goal ACT: 250 s, Reversal with protamine optional, Bivalirudin—not allowed?	Aspirin 81 mg/day indefinitely + Clopidogrel 75 mg/day × 90 days, If warfarin indicated (AF), then no clopidogrel
ACC/STS Recommendations [73]	–	UFH, Goal ACT: 300 s, Reversal with protamine recommended, Bivalirudin—not mentioned	Aspirin 81 mg/day indefinitely + Clopidogrel 75 mg/day × 3–6 months, If OAT indicated (AF), avoid triple therapy unless definite indication exists
CCS Statement [75]	–	–	Indefinite low-dose aspirin generally recommended + Thienopyridine × 1–3 months, If warfarin indicated (AF), then no clopidogrel
ACC VHD guidelines [51]	–	–	Clopidogrel 75 mg daily may be reasonable for the first 6 months after TAVR in addition to life-long aspirin 75 to 100 mg daily
ACCP VHD guidelines [30]	–	–	Clopidogrel 75 mg daily may be reasonable for the first 3 months after TAVR in addition to life-long aspirin 75 to 100 mg daily

*ACC* American College of Cardiology, *ACCP* American College of Chest Physicians, *AF* atrial fibrillation, *CCS* Canadian Cardiovascular Society, *OAT* oral anticoagulation therapy, *STS* Society of Thoracic Surgeons, *TAVI* transcatheter aortic valve implantation, *TAVR* transcatheter aortic valve replacement, *UFH* unfractionated heparin, *VHD* valvular heart disease

### Indian recommendation

- Antiplatelet agents either alone or in combination with anticoagulants are recommended after TAVI (antiplatelet agents long term) (grade A, EL 2).

### Bridging anticoagulants

The perioperative management of patients receiving VKAs or APAs and requiring a surgical or invasive procedure poses a significant dilemma for practising clinicians. One should take into account of different factors including the type of procedure, risk factors, and type, location, and number of heart valve prosthesis during the management of patients with mechanical heart valves in whom interruption of anticoagulation therapy is needed for diagnostic or surgical procedures.

To minimize the delay in achieving therapeutic anticoagulation, a “bridging” anticoagulant is prescribed. The “bridge” is administered parenterally [short-acting anticoagulant as unfractionated heparin (UFH) or low molecular weight heparin (LMWH)], thereby providing an immediate anticoagulant effect. However, the use of LMWH or UFH as perioperative bridging may be an off-label use as their use is not approved by regulatory authorities or drug manufacturers in this clinical setting as a bridging agent. There is a relative paucity of well-designed clinical trials to enlighten best practices; however, disproportionately large numbers of methodologically weak observational studies are available.

A study by *van* Geest-Daalderop JH et al. have evaluated days of interruption for acenocoumarol in patients undergoing invasive procedures. They found interruption for 2 days had lower bleeding risks compared to 3 days [76]. In patients undergoing major surgery or procedures, interruption of VKAs, in general, is required to minimize perioperative bleeding [77]. Tinker JH et al. have concluded that patients with cardiac valve prostheses and continuing anticoagulants have minimal risk when they stop the anticoagulant regimen for 1–3 days preoperatively and 1–7 days postoperatively [78]. A similar method was followed in a study with a small group of patients [79]. Perioperative bleeding and thromboembolic events in patients on anticoagulation with mechanical valves were evaluated in a prospective study. There were 72 complications observed in 603 interventions that resulted in an overall frequency of 11.9% (9.5%, hemorrhage and 2.5%, thromboembolism). Moreover, the level of anticoagulation was not associated with the occurrence of complications [80].

Moderate-intensity anticoagulant therapy (INR of 1.5 to 2.0) was reported to be safe and feasible for preventing thromboembolic complications in high-risk surgical patients who are receiving long-term OAT. The study included 18% patients with a mechanical valve [81]. However, VKA interruption may not be required in minor procedures like dental procedures [82, 83], minor dermatological procedures [84, 85], and cataract surgery [86]. A study has shown no significant difference in thromboembolic and major bleeding events between patients bridged with LMWH and those bridged with UFH [87]. The literature demonstrates that most studies assessing the use of LMWH as bridging anticoagulation have used therapeutic dose regimens [88]. Two studies have used low-dose LMWH (including patients with mitral valve prosthesis) [89, 90], but it is not clear if this is sufficient as it can be argued that higher doses of LMWH are needed for the prevention of arterial thrombosis. The latter, however, is not established. To aid decision making on bridging interventions, ACC and ACCP divide patients into high and low risk as per thrombotic risk stratification (Table 4).

**Table 4:** Thrombotic risk stratification as per ACC, ACCP, CSI and current guideline

Organizations	Category (% annual risk of thromboembolism)	Patient characteristics
ACCP [88] and CSI [10]	High risk (> 10%)	• Any mitral valve prosthesis • Any caged-ball or tilting disc aortic valve prosthesis • Recent (within 6 months) stroke/transient ischemic attack • Prior thromboembolism in temporary VKAs interruption • Types of surgery associated with an increased risk of stroke or other thromboembolism (e.g., cardiac valve replacement)
	Low risk (< 10%)	• Bileaflet aortic valve prosthesis without AF and no other risk factors for stroke
ACC [51]	High risk (> 10%)	• All patients with mechanical MVR or TVR, patients with an AVR and any risk factors (AF, previous thromboembolism, hypercoagulable condition, older-generation mechanical valves, LV systolic dysfunction (LVEF< 30%) / > 1 mechanical valve) for thromboembolism
	Low risk (< 5%)	• Bileaflet mechanical aortic valve and no other risk factors
Current guideline	High risk (> 10%)	• Going for any other major surgery irrespective of valve type and position • Any mitral valve prosthesis • Recent (within 6 months) stroke/transient ischemic attack • Prior thromboembolism in temporary VKAs interruption • Bileaflet mechanical aortic valve with risk factors • AF
	Low risk (< 10%)	• Bileaflet mechanical aortic valve and no other risk factors

*ACC* American College of Cardiology, *ACCP* American College of Chest Physicians, *AF* atrial fibrillation, *AVR* aortic valve replacement, *CHF* congestive heart failure, *CSI* Cardiological Society of India, *LVEF* left ventricular ejection fraction, *MVR* mitral valve replacement, *TVR* tricuspid valve replacement, *VKA* vitamin K antagonist

ACC and ACCP recommend uninterrupted VKA therapy with local hemostasis optimizing agents in procedures with minimal bleeding. These procedures include excision of basal and squamous cell skin cancers, actinic keratosis, and premalignant or cancerous skin nevi; dental cleaning, or simple treatment for dental caries (Table 5); surgery for cataracts or glaucoma. For tooth extractions and endodontic (root canal) procedures, ACCP recommends uninterrupted VKA with co-administration of an oral pro-hemostatic agent or stopping VKAs for 2 to 3 days (partial reversal). The patients should be informed of any minor bleeding (bleeding from gingival mucosa) and advised to continue any pro-hemostatic treatment given.

**Table 5:** Surgeries with high bleeding risks identified by ACCP [88]

• Urologic surgery and procedures consisting of transurethral prostate resection, bladder resection, or tumor ablation; nephrectomy; or kidney biopsy	
• Pacemaker or implantable cardioverter-defibrillator device implantation	
• Bowel resection, colonic polyp resection, typically of large (i.e., > 1–2 cm long) sessile polyps	
• Surgery and procedures in highly vascular organs, such as the kidney, liver, and spleen	
• Major surgeries such as cancer surgery, joint arthroplasty, reconstructive plastic surgery which could cause extensive tissue injury	
• Cardiac, intracranial, or spinal surgery	

*ACCP* American College of Chest Physicians

ACC recommends interrupting VKA for 2–4 days without bridging in patients with low thrombotic risks, i.e., bileaflet AVR without any risk factors (Table 6). ACC and ACCP recommend interruption of VKA with bridging anticoagulation in patients with any mitral valve prosthesis, any caged-ball or tilting disc aortic valve prosthesis, bileaflet AVR with additional risk factors, patients with recent (within 6 months) stroke or TIA, patients with prior thromboembolism during temporary interruption of VKAs. When interruption of VKA therapy is required, ACC recommends stopping 2–4 days before while ACCP recommends not less than 5 days before (considering 36–42 h as half-life for a complete reversal of anticoagulant action) the procedure. Both recommend restarting approximately 12 to 24 h after surgery (evening of or next morning) (Table 6).

**Table 6:** Recommendations on time frame for bridging anticoagulation

	ACCP [88]	ACC [51]	Current guideline
Interruption of VKA	Stop at least before 5 days and restart 12–24 h after surgery	Stop before 2–4 days restart 12–24 h after surgery	Stop before 2–4 days, after stopping VKA therapy, INR should be checked after 2 days and should be maintained < 2. Restart 12–24 h after surgery
Bridging with UFH or LMWW			
Stop VKA	Before at least 5 days	Before 2–4 days	INR should be checked after 2 days of stopping VKA therapy and next decision on therapy should be taken depending on INR value
Starting UFH or LMWH, when INR < 2	Not mentioned	48 h before planned surgery	
Stopping UFH or LMWH	4–6 h for UFH, 24 h for LMWH, before the procedure	4–6 h for UFH, 12 h for LMWH, before the procedure	
Resuming UFH or LMWH, when adequate hemostasis achieved	48–72 h	48–72 h	

*ACC* American College of Cardiology, *ACCP* American College of Chest Physicians, *INR* international normalized ratio, *LMWH* low molecular weight heparin, *UFH* unfractionated heparin, *VKA* vitamin K antagonist.

### Indian recommendation

- It is not recommended to interrupt VKA therapy during minor dental procedures (cleaning), dermatological procedures, and cataract surgery due to minimal bleeding (grade A, EL 2)
- In patients with low thrombotic risk (bileaflet AVR without atrial fibrillation and no other risk factors for stroke), it is recommended to interrupt VKA therapy without bridging (grade B, EL 3)
- Bridging anticoagulation is recommended in patients with high risk of thromboembolism during temporary interruption of VKAs (grade B, EL 3)
- When interruption of VKA therapy is required, it is recommended to stop for 2–4 days before the procedure. After stopping VKA therapy, INR should be checked after 2 days and maintained < 2. The VKA therapy should be restarted 12–24 h after surgery (grade A, EL 2)
- The reversal of VKA therapy during emergency surgeries can be achieved by administration of FFP. FFP requirement depends on the PT/INR value, liver function tests, and body weight of the patient (grade A, EL 2)
- Interruption of VKA therapy with bridging anticoagulants is recommended for not more than 2 days in case of elective major surgeries (grade A, EL 3)

#### Cardiac catheterization in patients with prosthetic valves

The Bridge or Continue Coumadin for Device Surgery Randomized Controlled Trial (BRUISE CONTROL) randomly assigned patients on warfarin undergoing implantation of a pacemaker or implantable cardioverter–defibrillator (ICD) to the continuation of warfarin or heparin bridging. The strategy of continued warfarin treatment compared to bridging with heparin markedly reduced the incidence of clinically significant device-pocket hematoma [91]. The COMPARE trial randomly assigned patients with AF undergoing catheter ablation to continued warfarin or discontinuation of warfarin with bridging. In this trial, patients randomized to continue warfarin had a lower risk of stroke and less bleeding [92]. In a randomized trial, patients on OAT referred for pacemaker or ICD were randomized to warfarin continuation versus interruption. There was a trend toward reduced complications in patients randomized to warfarin continuation, though the results were not statistically significant [93]. In another randomized trial, patients were assigned to either uninterrupted OAT or bridging with heparin. The study concluded that maintaining VKA was associated with significant reduction of in-hospital stay compared with bridging to heparin infusion [94].

A recent prospective observational study compared acenocoumarol continuation versus discontinuation in 489 patients undergoing trans-radial diagnostic catheterization. The study concluded that continuation of chronic OAT appears safe during trans-radial diagnostic catheterization [95]. Similarly, in a prospective study, Sanmartín M et al. concluded that the trans-radial approach appears to be a safe option and could be the technique of choice for patients continuing long-term acenocoumarol therapy as it eludes the problems and complications associated with the withdrawal of OAT [96].

The European Heart Rhythm Association (EHRA) position document on antithrombotic management in patients undergoing electrophysiological procedures recommends uninterrupted VKA in patients undergoing ablation procedures like pulmonary vein isolation (PVI) and in patients requiring implantation of cardiac implantable electronic devices; unless they are at very low risk for a thromboembolic event. EHRA recommends interruption of VKA without bridging with heparin in such patients. Furthermore, EHRA recommends not using the formerly commonly practised “bridging therapy” with UFH or LMWH since it significantly increases bleeding complications in this category of patients [97]. ACC recommends only slight modification in VKA dosing for procedures with a low bleeding risk, such as coronary angiography from the radial approach. With interventional procedures at higher risk, stopping VKA anticoagulation and using bridging therapy as is done for other surgical procedures have been recommended [51]. ACC and ACCP recommend considering an acceptable level of anticoagulation in a specific cardiac catheterization procedure.

### Indian recommendation

- Patients with low bleeding risk and undergoing coronary angiography with the radial approach may not require modification in VKA; however, in patients at high risk, bridging therapy is recommended (grade A, EL 1)
- VKA therapy may be reduced or withheld and bridging is considered for patients with the femoral approach in the electrophysiological procedure (grade A, EL 2)
- In patients who are at low risk of thromboembolic events, undergoing PVI, VKA dose should be adjusted to maintain INR < 2 and regular dose to be commenced after implantation (grade A, EL 2)
- In patients who are at very low risk of thromboembolic events undergoing cardiac implantable electronic devices, change in VKA therapy is not required; however, the dose should be titrated to achieve target INR < 2 (grade A, EL 2)

## Initiation, monitoring, and factors affecting anticoagulation

### INR testing

Many factors affect the level of VKAs and thereby resultant INR. The subtherapeutic target INR increases the risk of thromboembolic events, on the other side, above the therapeutic INR presents the patient to the risk of bleeding. INR monitoring helps in avoiding over coagulation and also assist in deciding the appropriate dosage regimen, Moreover, dangerous situations can be detected well in time with routine monitoring, which allows dose adjustment, as well as to take actions to prevent recurrence of such situations [98].

The most common test used to monitor anticoagulation therapy is the PT test. The PT is expressed as INR. The TTR is a good overall measure of the quality of antithrombotic treatment with VKAs in patients with VHD [99, 100]. It is recommended that the INR measurement should be performed in the National Accreditation Board for Testing and Calibration Laboratories (NABL) accredited laboratory. The laboratory should follow the clinical and laboratory standards institute guidelines for coagulation testing [101]. As per the guideline, the sample should be transported in a shortest possible time at an ambient temperature (15–22 °C) and the sample testing should be accomplished within 4 h of collection. As heparin has the potential to neutralize platelet releasates, testing for UFH monitoring should preferably be processed within 1 h. Extremes of temperature (i.e., both refrigerated and high) should be avoided. Transport delay might affect labile clotting factors such as FV and FVIII and lead to prolonged clotting times and in vitro loss of factor activity. In such cases, plasma should be separated and frozen and then transported [102]. With consideration of 4 h of sample travel time, a reliable INR facility should be available to the patient within 4 h travel time.

### Point-of-care INR testing

The gold standard for monitoring INR is the lab testing of blood obtained by venipuncture, in hospital. The point-of-care (POC) INR systems (coagulometer) can be an alternative to older laboratory testing of INR. POC monitors measure a thromboplastin-mediated clotting time, by an electrochemical mechanism, which is converted to plasma PT equivalent by a microprocessor and expressed as either the PT or the INR. POC testing involves putting a single drop of blood from a finger stick, onto a test strip. It is aimed at convenience for the patient, faster test results to a healthcare provider, faster decision making, improved clinical outcome, and reduced healthcare resources. Lucas F et al. validated a novel whole-blood capillary technique for measuring the PT for the first time, and subsequently, this technique became available for professional use in doctor’s offices and hospitals [103]. A number of instruments for home anticoagulant monitoring have been developed later. Usage of the initial meters was largely unreliable due to considerable variations and limited quality checks and they had relatively poor precision and did not use whole blood calibrators. However, the POC devices differ in the method of endpoint detection and use microfluidic technology. McBane RD et al. compared two commercially available POC devices, Coaguchek and ProTime 3 in determining the INR and found that correlation with plasma was greater for the Coaguchek (*r*^2^ = 0.90) compared with the ProTime 3 device [104]. Furthermore, the INR differed from the laboratory values with an over-estimation on the lower end of INR values. With the advent of newer technology for measurement and quality control procedures, the reliability of PT/INR meters have vastly improved. The cost of POC meters ranges from 5000 to 10,000 rupees with the cost of the strip ranging from 50 to 100 rupees. This is higher than the 30–40 rupees that the laboratory testing costs [105]. However, these devices are economic as they reduce the cost of visiting the healthcare facility. This is of great importance in India, as most of INR facilities are available far in the urban or semi-urban area. These POC devices have shown to be cost-effective for patients on long-term anticoagulants [106, 107]. Studies have found a statistically significant advantage of self-management methods in achieving the good INR control in patients with mechanical valves [108, 109]. By comparing the outcomes of self-monitoring or self-management of OAT with standard monitoring in 8950 patients, a recent Cochrane database systematic review (28 RCTs) reports that participants who self-monitor or self-manage can improve the quality of their OAT with reduction of thromboembolic events [110]. Sharma P et al. in their systematic review (26 RCTs, 8763 patients) detected the clinical and cost-effectiveness of POC tests (CoaguChek system, INRatio2 PT/INR monitor and ProTime Microcoagulation system) for the self-monitoring of the coagulation status of people receiving long-term VKA therapy. The study report that self-monitoring, and in particular self-management with POC device, of anticoagulation status appeared cost-effective when pooled estimates of clinical effectiveness were applied [111]. A study by Lakshmy R et al. has shown the reliability and accuracy of Coaguchek XS INR kits in Indian patients [105].

ACCP and ACC/AHA recommend the practice of self-management of patients over outdoor INR monitoring for VKA anticoagulation, in patients who are motivated and can demonstrate competency in self-management strategies [112, 113].

### Indian recommendation

- The practice of patient self-management (PSM) of anticoagulation with patient self-monitoring is recommended (grade B, EL 1)
- Use of POC devices can be alternated with the conventional laboratory testing to reduce hospital visits and the cost of the treatment (grade B, EL 1)

### Loading dose, dose adjustments, and frequency of INR monitoring

There is wide variation in patient response to VKA dose. In view of this and narrow therapeutic index, VKA dose should be optimized for initiation of therapy. Still, there is a considerable uncertainty about loading dose of VKAs. Moreover, there are no randomized data that address the optimal time to start anticoagulation therapy; however, all major guidelines recommend that VKA therapy should be started in the first 24–48 h after the surgical procedure.

The 2 mg initial dosing of acenocoumarol has been investigated by Amian A et al. and found that it is as effective as the 4 mg dose in reducing thromboembolic events and number of hemorrhage episodes [114]*.* Harrison L et al. report less excess anticoagulation with a 5 mg loading dose of warfarin than 10 mg loading dose; the smaller dose also avoided the development of a potential hypercoagulable state caused by precipitous decreases in levels of protein C during the first 36 h of warfarin therapy [115]. Crowther MA et al. have also demonstrated that 10 mg loading dose of warfarin is not more effective than a 5 mg loading dose in achieving an INR of 2.0 to 3.0 by day 4 or 5 of therapy [116]. Garcia D et al. found a decrease in warfarin dose requirements with age. The authors suggested to lower the empiric starting dose of 5 mg daily and maintenance doses in the geriatric age group to avoid over-anticoagulation [117]. In a study by Van Geet-Daalderop JH et al., a model of standardized individualized dose regimen was evaluated. The model proposed an initial dose of 6–4–2 mg acenocoumarol, exceeding this mean daily dose to a small extent, was appropriate for patients younger than 70–75 years. An initial dose regimen of 4–2–1 mg was suggested in the elderly patients [118]. A meta-analysis by Mahtani KR et al. found considerable uncertainty between the use of a 5 mg and a 10 mg loading dose for the initiation of warfarin. There was some evidence of lower initiation doses or age-adjusted doses in elderly patients leading to fewer high INRs. However, there was insufficient evidence to warrant genotype-guided initiation [119]. Similar findings were also observed in another meta-analysis by Heneghan et al. [120]. Lastoria S et al. compared 5 mg versus 10 mg loading dose. The 10 mg dosage regimen took less time to attain the TR and was associated with lower warfarin doses at discharge and better INR at first out-patients follow-up visit [121]. A two-step dosing nomogram has been prospectively evaluated by Kim YK et al. [99]. Moreover, Dhanya PS et al. have found no difference in TTR achieved by warfarin versus acenocoumarol [39].

When VKA therapy is initiated, the INR may begin to respond after 2 to 3 days because of the depletion of factor VII. Bridging anticoagulation by UFH or LMWH is recommended by ACC, ACCP, and Cardiological Society of India (CSI) during this initial period, and continued until the INR has been in the TTR for a minimum of 24 h [10, 51, 88]. ACC and CSI recommend an initial dose of warfarin as 5 mg. However, individualization of warfarin dose depending on various factors (e.g., age, bleeding risk, medication compliance history) and anticipated drug interactions is also recommended. Lower initiation dosages might be required in older patients and persons with liver disease, poor nutritional status, or heart failure (HF). The ACCP guidelines suggest an alternative warfarin initiation dosage of 10 mg daily for the first 2 days of therapy, in addition to a 5 mg initial dose of warfarin, in healthy persons who can be treated as outpatients [122]. These recommendations were based on a meta-analysis of clinical trials which included venous thromboembolism (VTE) patients. They also compared early start (day 1 or 2 of heparin) versus late start (days 3–10 of heparin) for the VKA therapy together with UFH or LMWH therapy and recommended to start on day 1 or 2 of heparin.

As per the current ACCP recommendations, the first INR after baseline INR can be obtained after the patient has received two or three doses. Then, the frequency could be decreased to twice weekly until the INR is within the TR. Later the monitoring could be carried out every weekly, followed by every other week, and finally monthly. Moreover, the guidelines allow considering INR monitoring up to every 12 weeks in patients who are stable (defined as having at least 3 months of consistent results with no need to adjust VKA dosing). However, the frequency of monitoring should be increased if a patient’s INR becomes subtherapeutic or supratherapeutic, and reduce when INR is stabilized [122]. For patients taking VKAs with previously stable therapeutic INRs who present with a single out-of-range INR of ≤ 0.5 below or above therapeutic INR, ACCP suggests continuing the current dose and testing the INR within 1 to 2 weeks without routinely administering bridging heparin.

### Indian recommendations

- The initial recommended dosage of VKAs is warfarin 5 mg and acenocoumarol 2 mg. However, individualization of VKA dose depending on age, bleeding risk, medication compliance history, and anticipated drug interactions is also recommended (grade A, EL 2).
- After baseline INR is determined, the next INR can be obtained after the patient has received two or three doses. Then after, the frequency of INR monitoring should be decided according to the value of INR (grade C, EL 4).
- INR checking should be as follows: first, at discharge; second, 48 h; third, after 1 week of discharge; then after the frequency should be decided depending on INR value at first week (grade C, EL 4) (PT/INR reports 48 h after discharge as patient will be in the loading phase at the time of discharge. The fluctuation of INR for a week before the revisit after discharge not desirable).
- In patients with stable INR for 3 months with no dose adjustment, it is recommended to consider INR monitoring in every 12 weeks (grade A, EL 1). However, in the case of subtherapeutic or supratherapeutic INR, the frequency of monitoring should be increased until it stabilizes again. It should be monitored 1–2 monthly in high thrombotic risk patients (grade C, EL 4).
- In most asymptomatic fluctuations, the dose adjustment should be done by calculating the weekly or monthly dose of VKAs (grade C, EL 4).
- For patients taking VKAs with previously stable therapeutic INRs who present with a single out-of-range INR of ≤ 0.5 below or above therapeutic INR, it is recommended continuing the current dose and testing the INR within 1 to 2 weeks with no routinely administering bridging with heparin (grade A, EL 2).
- If INR falls below the target in high-risk patients, an increase in VKA dose or UFH may be considered (grade C, EL 4).

### Proposed algorithm/guide

**Fig. 1:**
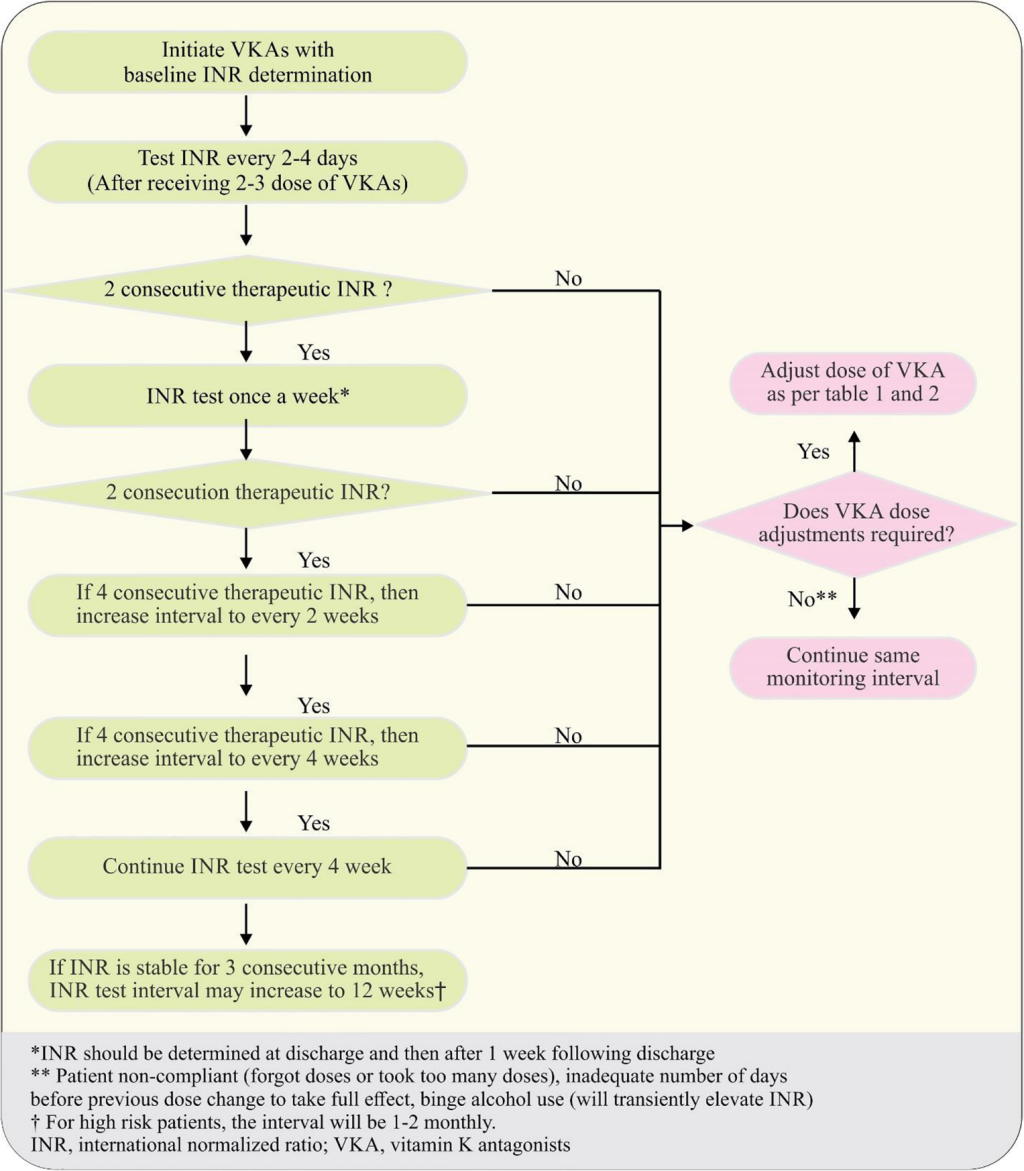
Proposed initiation and maintenance of VKA therapy

A stepwise guide for initiation and maintenance of VKA therapy is as follows:

1. Patient consent
2. Anticoagulation with heparin
3. Start VKAs

1. Initiation of VKAs

- Establishment of baseline INR should be done in every case, which will guide further therapy.
- Initial dose: initial dose of warfarin is typically 5 mg/day (acenocoumarol 2 mg/day) in most patients. A reduced dose may be considered for patients > 70 years of age, elevated baseline INR (> 1.1), hypo-albuminemia patients (e.g., malnourished, liver disorders, postoperative), impaired nutrition (weight < 45 kg), HF, taking medications that increase the sensitivity of VKAs, or previously documented increased sensitivity to VKAs.
- Whenever feasible, a single strength VKA tablet should be prescribed such that doses are multiples of one tablet strength. Patients should take their VKA once a day at the same time in the evening, and have their INR test performed in the morning. This limits diurnal variations and provides the physician with the same day window for dosage adjustment in the event of an unanticipated INR change.

2. Check INR on morning of day 2 and adjust the dose-proposed nomogram
3. Stop heparin when INR is therapeutic
4. Regular INR checks
5. INR target and frequency of monitoring

- It is recommended that during the initiation phase, INR should be monitored every 2–4 days, until INR is in the TTR for two consecutive values. Once stabilized, INR should be monitored weekly. The interval can be gradually increased up to every 4 weeks if the INR remains stable and within TR. However, ACCP recommends monitoring of INR every 12 weeks in the stable patients. The monitoring frequency should be increased with any substitution, deletion, or addition of any drug as a concomitant therapy during OAT. The INR testing interval is presented in the flowchart as below (Fig. 1).

6. Maintenance therapy

- Dosage adjustment is not required for minor fluctuations of INR as long as the results remain within the patient’s target range. Fluctuations of INR beyond the patient’s target range should always prompt a direct communication with the patient to determine the cause. Consider causes such as a change in dosage of VKAs, patient compliance, medications including over the counter (OTC) drugs, dietary changes, unusual alcohol consumption, or intercurrent illness.
- The recent trend is to change the total VKA dose for example if the patient is taking 5 mg/day, the weekly dose is 35 mg. If the dose must be decreased by 10%, then the weekly dose should be 35 mg − 3.5 mg = 31.5 mg and the daily dose becomes 31.5 mg ÷ 7 = 4.5 mg.

**Fig. 2:**
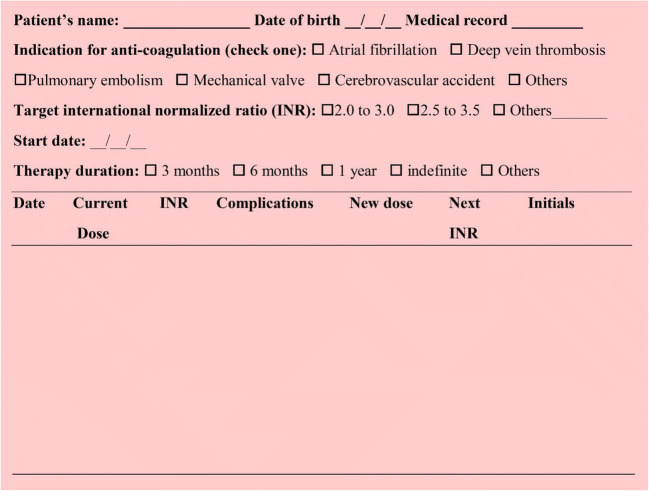
A representation of outpatient anticoagulation flow sheet

Once a patient makes the transition from the initial dosing phase to the maintenance phase, more consideration to the multiple factors that may affect the INR should be given when interpreting low or high INR values. The ideal regimen should provide the same dose every day, but this is not always possible. VKAs come in many tablet strengths: 1, 2, 2.5, 3, 4, 5, 6, 7.5, and 10 mg. Still, for some patients, a given tablet strength might not be enough while the next higher tablet strength may be too much. In this situation, one needs to give different doses on different days of the week. It is better if the doses are similar rather than greatly different. In most cases, alternating doses (e.g., 2.5 mg alternating with 5 mg) or repeating doses (e.g., 2.5 mg, then 2.5 mg, then 5 mg) should be avoided, as they provide different total weekly doses of VKAs. The algorithm can be prepared as shown in Fig. 1 (and associated tables, Table 7 [123]: [warfarin nomogram] and Table 8 [124] [acenocoumarol nomogram for dosage adjustments]; adapted from that of the anticoagulation service at the University of Michigan and is consistent with recommendations from the ACCP guideline). The proposed algorithm for initiation and maintenance of VKA is described in Fig. 1. To help the physician in selecting the number of tablets of given strength of coumadin based on the availability of that tablet strength, a computer-based programme can be used (Fig. 2) [125, 126].

**Table 7:** Proposed nomogram for dose adjustment for patients on warfarin maintenance therapy; target INR 2.0–3.0 or 2.5–3.5, no significant bleeding [123]

INR	Intervention (refer to flowchart in Fig. 1 for timing of next INR)
≤ 1.5	• Give one-time top-up equal to 20% of weekly dose and increase weekly dose by 10–20%
1.5 < INR < therapeutic range	• No change in dose • If two consecutive INRs are low, increase weekly dose by 10–20%
INR in therapeutic range	• No change
INR > therapeutic range but < 5.0	• Lower weekly dose (10–20%) or consider omitting one single dose • Increase the frequency of INR monitoring and resume therapy at 10–20% lower weekly dose when INR therapeutic Note: If the INR is only minimally elevated (0.1–0.4 above upper limit of the therapeutic range), dose reduction may not be necessary
INR 5.0–9.0	• Omit 1 to 2 doses then recheck INR • Increase the frequency of INR monitoring and resume therapy at 10–20% lower weekly dose when INR therapeutic • If the patient is at high risk of serious bleeding, consider administering vitamin K 1 to 2 mg orally
> 9.0; no bleeding	• Discontinue warfarin temporarily, consider administering vitamin K 2–5 mg orally then recheck INR • Increase the frequency of INR monitoring and resume therapy at 20% lower weekly dose when INR therapeutic • Give additional vitamin K if INR is not substantially reduced by 24 h.

*INR* international normalized ratio

**Table 8:** Proposed nomogram for dosage adjustments for patients on acenocoumarol maintenance therapy; target INR 2.0–3.0 or 2.5–3.5, no significant bleeding [124]

INR	Intervention (refer to flowchart in Fig. 1 for timing of next INR)
< 1.3	Add 1 mg/day to the current dose and repeat INR after 1 week
1.4–2.0	Add 0.5 mg/day to the current dose and repeat INR after 1 week
2.1–3.5	Maintain current dose
3.6–4.0	Decrease current dose by 0.5 mg/day and repeat after 1 week
> 4.0	Stop the drug for 3 days and repeat INR till INR falls. If INR < 4.0 on repeated measurement, then to follow as above.

*INR* international normalized ratio

### Factors affecting VKA therapy

#### Pharmacogenetics

Growing evidence indicates that up to 60% of the individual pharmacological response to warfarin might be due to genetic variables and affected by polymorphisms in the genes mainly vitamin K epoxide reductase complex subunit 1 (*VKORC1*), the target enzyme of warfarin, and cytochrome P450 2C9 (*CYP2C9*), the main enzyme involved in warfarin metabolism [127]. Although the genotypes of *VKORC1* and *CYP2C9* are clearly the most important genetic factors for warfarin response, genetic variations in other genes, for instance, *CYP4F2* and *GGCX* also show significant association with warfarin response [127]. Compared to *CYP2C9* polymorphisms, *VKORC1* polymorphisms have been reported to be more potent modifiers of warfarin response. Individuals with *VKORC1 A* haplotype (H1 and H2) have been shown to require low warfarin dose compared to individuals with *VKORC1 B* haplotype (H7, H8 and H9) due to decreased expression of messenger ribonucleic acid [128]. *VKORC1*-*1639G* > A (rs9923231) SNP is a good predictor to distinguish high and low dose requirement group over the *VKORC1* haplotype [129–131].

Warfarin dose titration algorithms have been established which can provide a base for genetic testing in India [132, 133]. Many of the studies have earlier shown that around 75–80% of the Indian population carries *VKORC1*-*1639GG* (rs9923231) genotype, which is associated with high dose requirement [129, 134–136]. A study by Rathore S et al. has shown that the *VKORC1*-*1639 G > A* status can be indicative of establishing the therapeutic dose of OATs in Indian prosthetic valve patients [136]. When inter-individual dose variability of acenocoumarol in the Indian patients with *CYP2C9* and *VKORC1* gene polymorphisms were evaluated, lower dose requirements for carriers of the studied polymorphisms had been identified and there was considerable variability in the dose requirements of carriers and non-carriers of the variant alleles [137]. Similarly, Krishna Kumar D et al., by studying South Indian patients with heart valve replacement, report decreased requirement of the daily maintenance dose of acenocoumarol in patients with *CYP2C9* and *VKORC1*(-*1639 G* > A) genetic polymorphisms [138]. Although, translation of warfarin pharmacogenetics into clinical practice is slowly emerging in India.

Recently, FDA has recommended pharmacogenetics testing before initiating warfarin. However, ACCP recommends against the routine use of pharmacogenetic testing for guiding doses of VKA in patients initiating VKA therapy. CSI has mentioned about the availability of tests but has not recommended routine test for initiation of warfarin [9, 113].

### Indian recommendation

- If the patient did not achieve target INR in spite of high dose of warfarin, pharmacogenetics testing may be considered, provided that it is easily available and cost-effective (grade C, EL 4).

#### Anticoagulant interactions

##### Drug-drug interaction

As VKAs are metabolized mainly by CYP2C9, the inhibition of CYP2C9 results in a decreased catabolism of VKAs and a stronger anticoagulant effect, whereas its induction enhances their catabolism and leads to a lower anticoagulant effect.

Amiodarone, fluconazole, fluvastatin, fluvoxamine, isoniazid, lovastatin, miconazole, metronidazole, trimethoprim-sulfamethoxazole, and phenylbutazone are known inhibitors of CYP2C9, thus potentiating VKAs effect [139]. Antibiotics, which cause decreased production of vitamin K by intestinal microbiota result in an increased sensitivity to VKAs. Barbiturates, namely carbamazepine and rifampicin, are inducers of CYP2C9 [139]. Other drugs interact with other cytochromes also involved in the metabolism of VKAs, such as quinolones which inhibit CYP1A2, and macrolide antibiotics which inhibit CYP3A4 [139].

In the Apixaban for Reduction in Stroke and Other Thromboembolic Events in Atrial Fibrillation (ARISTOTLE) trial, patients receiving both warfarin and amiodarone had time in the TR lower than patients not on amiodarone (56.5% vs. 63.0%; *p* < 0.0001) [140]. OTC medications, i.e., paracetamol and other nonsteroidal anti-inflammatory drugs (NSAIDs) were shown to enhance the anticoagulant effect of warfarin, thus patients receiving warfarin must monitor their INR more frequently when taking these medications, especially paracetamol at doses exceeding 2 g/day. This interaction has been demonstrated in placebo-controlled RCTs. Patients on stable OAT with warfarin were randomized to receive placebo or paracetamol 2 g daily or 3 g daily for 10 days. Paracetamol (2 g/3 g daily) was found to potentiate the anticoagulant response produced by warfarin [141]. A large number of drugs interfere with VKA. In a study by Wittkowsky AK et al., it was shown that 81.6% of the 134,833 patients receiving long-term warfarin therapy were prescribed a concurrent prescription for at least 1 potentially interacting drug, with interactions known to increase the INR [142]. Warfarin drug interactions were one of the common drug interaction found in a cardiology ward in one prospective study conducted in India [143].

ACCP suggest avoiding concomitant treatment with NSAIDs, including COX-2-selective NSAIDs, and certain antibiotics for patients taking VKAs [113].

Although not a direct interaction but dehydration due to aggressive diuresis postoperatively and fluid restriction increases INR and dosage of warfarin has to be adjusted or stopped. If vomiting or diarrhea precipitates dehydration, it can derange INR and monitoring of INR and alteration of Warfarin dosage is warranted.

### Indian recommendation

- It is recommended to avoid drugs that inhibit or enhance the activity of cytochrome P450 during the VKA therapy (grade A, EL 1).
- It is recommended to educate patients on drug interaction with over the counter drugs and antibiotics, and need for frequent monitoring INR when they are used (grade B, EL 3).
- Amiodarone, if needed, should be used with caution along with VKA therapy (grade A, EL 1).
- NSAIDs should not be used with VKAs, if at all NSAIDs are needed, paracetamol may be considered with close supervision (grade A, EL 1).

#### Drug-herbs interaction

The interaction between VKAs and herbs is well reported in the literature. These include the interaction with *Panax ginseng*, *Hypericum perforatum*, Salvia milthiorizza, Gingko biloba, Sereno arepens, Angelica sinensis, Vaccinium species, *Allium sativum*, *Zingiber officinale*, *Tanacetum parthenium*, Lucium barbarum, *Matricaria chamomilla*, *Boswellia serrata*, and *Camellia sinensis* [144, 145].

##### Drug-food interaction

Indians with their different dietary habits compared to their western counterparts are more prone to VKA-food interactions. Indian takes more of dietary green leafy vegetables which would prevent the achievement of target INR on patients with VKAs and cause variability in INR values.

The interaction between the dietary vitamin K and VKAs is well known. Evidence has demonstrated that anti-coagulated patients should maintain a steady intake of vitamin K once INR stability has been achieved. Foods such as mango, grapefruit juice, cranberry, caffeine, and alcohol potentiate the effect of VKAs, whereas vitamin K-rich foods like dark green and cruciferous vegetables, animal products and seafood, soybean oils, fenugreek, soy milk, and green tea inhibit the effect of VKAs. Patients should be educated not to avoid vitamin K-rich foods; however, they should be advised to maintain the vitamin K content in the diet constantly. The best way to achieve this is by avoiding changes to normal eating patterns [146, 147].

In special situations, such as during fasting, the VKA therapy should be closely monitored in patients who are on long-term anticoagulant therapy. Lai YF et al. in their prospective study evaluated the effects of fasting in patients taking warfarin. The study reported that the mean INR of medically stable patients taking warfarin increases significantly with fasting. Moreover, closer monitoring or a dosage adjustment may be necessary during fasting in patients with higher end of INR target ranges or at increased risk of bleeding [148]. Similarly, a recent prospective study also suggested for closer follow-up of fasting patients who are on warfarin therapy [149]. Moreover, a pilot study also demonstrated that fasting deeply affects the stability of INR and increases the risk of bleeding in patients treated long-term with acenocoumarol [150].

ACCP suggests against routine use of vitamin K supplementation (food) for patients with VKA. CSI suggests avoiding vitamin K-rich food [9, 113].

### Indian recommendation

- Patients are advised to maintain constant vitamin K composition in their diet to avoid fluctuation of VKA therapy (grade C, EL 4).
- Patient in special situations such as fasting, VKA therapy should be carefully monitored (grade B, EL 2).

### Anticoagulation in special patient populations

#### Pregnancy

Increase in fibrinogen factors VII, VIII, and X; von Willebrand factor; and a relative decrease in protein S activity, stasis, and venous hypertension during pregnancy results in the hypercoagulable state [151]. This hypercoagulable state extends into the postpartum period too and requires a persistently higher maintenance dose of VKAs [152]. The increase in total blood volume affects the distribution of anticoagulants in pregnancy contributing to unpredictable changes in the amount of medication required [152, 153].

Optimal anticoagulation therapy is essential for pregnant patients; however, the appropriate choice of agent among the existing options (VKA, UFH, or LMWH) is highly debatable [151]. The warfarin enables it to cross placental barriers and cause embryopathy. The risk is high in organogenesis phase of first 6–9 weeks of gestation. The overall risk of embryopathy is < 10% in the first trimester and it reaches up to the level of the non-treated population after second trimester. The embryopathic actions are dose-dependent; reduced adverse events are seen with dose < 5 mg [154–156]. Warfarin can be replaced with LMWH since they do not cross the placenta. However, use of heparin is associated with the reported incidence of 12–24% of the increased risk of maternal thromboembolic events [157].

According to a published review, it is very common in India that pregnancy is diagnosed late in the first trimester. Furthermore, uneducated patients and who have no idea about the teratogenic effects, the risk of embryopathy increases with continuous use of VKAs in the first trimester. Women of childbearing age should be warned about the teratogenic and harmful effects of VKAs, especially in early pregnancy. They should be advised to use secure methods of contraception while on VKAs. In case of suspected pregnancy, early tests 5 weeks from the last menstrual period must be offered. In India, enforcing the practice of conversion to therapeutic once-daily LMWH prior to conception is difficult. Furthermore, all newer anticoagulants are contraindicated in pregnancy and lactation [158].

In a prospective study, women with mechanical heart valves were assigned to receive warfarin throughout pregnancy or subcutaneous heparin in the first trimester and warfarin for the other trimesters. Both groups received heparin at the time of delivery. There were no warfarin-induced fetal malformations. There were eight minor thromboembolic episodes and one prosthetic valve thrombosis (PVT) and one maternal death, not different between the groups. The incidence of spontaneous abortion was similar between the groups. The authors recommend the use of warfarin in the first trimester of pregnancy [159]. Likewise, a 10-year patient experience study suggests that continued oral intake of anticoagulants is safe and successful in pregnant patients with mechanical heart valve prosthesis [160]. A retrospective study to analyze the maternal and perinatal outcome in women with prosthetic heart valves on different anticoagulant regimens (heparin, warfarin, and acenocoumarol) reveal that no anticoagulant regimen is entirely safe during pregnancy as there is a certain degree of risk with each regimen [161]. Similarly, another study based on 25 years of anticoagulation experience in pregnancy also showed no anticoagulant regimen is entirely safe in pregnant women; heparin did not offer a clear advantage over OAT [162]. A simplified guidelines, developed by Panduranga P et al., for anticoagulation management in developing countries where factor Xa measurement facility is not available in hospital suggest that the anticoagulant treatment suitable for pregnant women with mechanical valve prosthesis is dependent on the availability of anti-factor Xa level monitoring facilities, the patient’s pre-pregnancy dose of warfarin, and the type of anticoagulant preferred by the patient in relation to the maternal and fetal risks [163]. Moreover, the guidelines also recommend close monitoring of the patient by a team of healthcare practitioners, including cardiology, hematology, and obstetric specialists. The current ACC, ACCP, CSI guidelines recommend warfarin in first trimester if dose required for target INR is < 5 mg. The warfarin should be replaced by heparin at 36 weeks of gestation before delivery (Table 9).

**Table 9:** Recommendations on management of anticoagulation in pregnancy

Before 36 gestational weeks		
Oral VKAs	ACC/AHA [51]	• Can be used throughout pregnancy (class IIa) with substitution by UFH/LMWH during weeks 6–12 of gestation if 1) Preferred by the patient 2) The dose of warfarin required to achieve target INR > 5 mg (class IIa) • Just before planned delivery has to be replaced by UFH or LMWH (class I) • Strongly recommended in MPV in second and third trimesters
	ACCP [164]	Can be used throughout pregnancy in high risk^a^ patients with UFH or LMWH before delivery (grade 1A)
	ESC [165]	If warfarin daily dose is < 5 mg, oral anticoagulation is the safest regimen throughout the pregnancy
	CSI [10]	Patients requiring smaller doses (< 3 mg acenocoumarol,< 5 mg warfarin) should be maintained on oral VKA
Heparin derivatives	ACC/AHA [51]	• 1) SC use of LMWH and UFH throughout pregnancy, 2) SC UFH in the first trimester completely removed in current 2014 guidelines • The patients who prefer: ^b^LMWH administered twice daily and the dose should be adjusted to attain peak anti-factor Xa levels: 0.8–1.2 U/mL approx. 4–6 h after the injection. Alternatively, continuous IV UFH (with aPTT at least twice that of the control) during the 1st trimester is permissible if warfarin dose > 5 mg/day • IV UFH in the first trimester is difficult from a practical standpoint, as 3-month hospital admission required
	ACCP [164]	• Monitored UFH/LMWH can be option throughout gestation or during 6–12 weeks of gestation (grade 1A) • In low-risk patients, LMWH should be given twice daily with anti-factor Xa levels of 0.35–0.70 U/mL 4 h after SC injection • The starting dose should be 100 U/kg of dalteparin and 1 mg/kg of enoxaparin.
	ESC [165]	• LMWH and UFH considered if warfarin dose > 5 mg • LMWH adjusted to achieve anti-factor Xa activity of 0.8–1.2 U/mL 4–6 h after administration, monitored on a weekly basis
	CSI [10]	• Patients with VKA (> 3 mg acenocoumarol, > 5 mg warfarin) should be considered for heparin therapy • Switch over to heparin from before conception/from 6th week is widely used, but not always needed • LMWH therapy requires monitoring of factor X assays
	All of the above guidelines agree that LMWH should be given twice daily and that it is harmful to administer LMWH without regularly monitoring the patient’s anti-factor Xa levels	
Aspirin	ACC/AHA [51]	Low dose (75–100 mg/day) given in second and third trimesters (class I)
	ACCP [164]	Low dose is given in addition to anticoagulation in high-risk patients (grade 2C)
	ESC [165]	Not recommended
	CSI [10]	Stable patients requiring high doses of VKA, the target INR may be lowered by 0.5 and aspirin added for the first trimester to avoid heparin
Target INR	ACC/AHA [51]	3 for all prosthetic valve patients
	ACCP [164]	2–3 for patients with bileaflet aortic valves w/o high-risk factors^a^
	ESC [165]	No target recommendation
	CSI [10]	Not mentioned
After 36 gestational weeks		
ACC/AHA [51]	• Stopping warfarin at 36 weeks and starting continuous IV UFH with aPTT monitoring, which should be continued until approximately 2–3 weeks before the planned delivery • Additionally, UFH can be discontinued 4–6 h before the planned delivery and restarted 4–6 h after delivery • In the absence of significant bleeding, oral warfarin should then be initiated 24 h after the birth	
ACCP [164]	• Stopping warfarin at 36 weeks and starting dose-adjusted IV UFH or LMWH • This treatment should continue until 36 h before delivery when LMWH should be replaced by IV UFH	
ESC [165]	• Continuing VKA until the patient is close to term^c^ (At this point, warfarin should be replaced by UFH/LMWH • If spontaneous labour occurs on oral anticoagulation, cesarean section is indicated due to obstetric-related causes	
CSI [10]	• If cesarean section is planned, VKA discontinued 3 days before • If vaginal delivery is expected VKA discontinued at 34 weeks and LMWH with monitoring of factor X assays. Labor should be initiated at 36–37 weeks • If labor starts unexpectedly, 2–4 mg IV vitamin K and FFP administered to reduce the risk of fetal injury. VKA restarted on day 1–2 with usual maintenance dose when hemostasis is adequate	

*ACC*/*AHA* American college of cardiology/American heart institute, *ACCP* American college of chest physician, *aPTT* activated partial thromboplastin time, *ESC* European society of cardiology, *INR* international normalized ratio, *IV* intravenous, *LMWH* low molecular weight heparin, *MPV* mechanical prosthetic valves, *SC* subcutaneous, *UFH* unfractionated heparin, *VKA* vitamin K antagonists

^a^First generation prosthesis, mitral valve prosthesis, history of thromboembolism, atrial fibrillation, left ventricular dysfunction

^b^ACC/AHA recommends discussing the preferred option of anticoagulation in pregnant patients

^c^Although the word term is not specified, it is generally accepted to signify 48 h before delivery

### Indian recommendation

- Women with valve replacement should be advised to plan their pregnancy and inform the surgeon if the period is missed (grade C, EL 4).
- Women of childbearing age should be warned about the teratogenic and harmful effects of VKAs, especially in early pregnancy (grade C, EL 4).

#### Before 36 weeks of gestation

- Oral VKA therapy is recommended throughout pregnancy in patients with daily warfarin dose requirement of ≤ 5 mg (or equivalent acenocoumarol dose) with target INR of 3 (grade B, EL 3)
- Subcutaneous UFH with activated partial thromboplastin time (aPTT) monitoring should be considered if warfarin dose is > 5 mg (or for equivalent acenocoumarol dose) (grade B, EL 3).
- Low-dose (75–150 mg) aspirin is recommended in second and third trimester (only in high-risk patients or all patients or in stable patients to reduce INR by 0.5) (grade C, EL 4).

#### At 36 weeks of gestation

- If the patient is hospitalized, VKA may be substituted with UFH. If not, VKA therapy should be discontinued prior to admission for delivery. (grade C, EL 4).
- When hemostasis is adequate, VKA therapy should be restarted on day 1 at maintenance dose along with heparin. (grade C, EL 4).

#### Elderly population

Anticoagulation management in elderly patients is a challenging issue. Indeed, elderly patients are at high risk of thromboembolic and hemorrhagic events. The benefit-risk balance of anticoagulation therapy should be carefully assessed when decisions are made about introducing and/or continuing the treatment in an individual elderly patient. Specific considerations, discussed below, need to be taken into account to maximize the safety of anticoagulation in the elderly [166, 167].

##### Comorbidities and co-medication

Elderly patients are more likely to have comorbidities and therefore multiple prescriptions, this is a key point to consider when introducing anticoagulation in this population of patients. This is particularly true for VKAs because these drugs have a narrow therapeutic index and multiple pharmacokinetic and pharmacodynamics potential alterations [166]. In a cross-sectional study by Rouaud A et al., the impact of comorbidities on quality of INR control in geriatric population was analyzed [168].

##### Pharmacokinetics in the elderly

An age-related decrease in body weight seems to affect VKAs’ pharmacokinetics. The change in the plasma proteins and other cellular components may modify the unbound heparin levels leading to unpredictable pharmacokinetics [169].

##### Pharmacodynamics in the elderly

Poor dietary vitamin K intake among elderly is one of the major factors contributing to variability of response and greater sensitivity to VKAs which leads to a reduced competitive antagonism to the effect of VKAs [166]. On the contrary, vitamin K containing OTC multivitamin tablets can significantly reduce response to VKAs [170]. Other mechanisms involved in increased sensitivity to VKAs are decreased production of vitamin K by intestinal flora in the presence of broad-spectrum antibiotics or increased catabolism of vitamin K-dependent clotting factors in hypermetabolic states such as fever [171, 172]. The concomitant intake of medications interfering with platelet aggregation, such as aspirin or NSAIDs, frequently prescribed in elderly patients, increase the bleeding risk [173].

##### Hemorrhagic risk

Bleeding and especially intracranial hemorrhage is the most dreaded complication of anticoagulant therapy. Regardless of the category of anticoagulant, increasing age represents an independent risk factor for bleeding with anticoagulation in the TR [174]. Insufficient education has been shown to be a major risk factor for anticoagulation-related bleeding complications in the elderly [175].

##### Renal function

The most significant change in organ function affecting drug pharmacokinetics is the decline in renal function, with an average loss in glomerular filtration rate (GFR) of 0.75 mL/min/year. Hence, to avoid adverse effects related to the excessive accumulation of drugs excreted by the kidney, a routine estimation of renal function is recommended in all geriatric patients [176]. VKAs are metabolized in the liver while other anticoagulants depend on renal excretion. Thus, VKAs do not require dosage adjustments in renal impairment patients and offer convenience in the elderly population.

The European Society of Cardiology (ESC) working group on thrombosis recommends considering all of the above factors for antithrombotic therapy in elderly [10, 177].

### Indian recommendation

- Frequent renal tests and observation for adverse effects with concomitant medications are recommended in elderly patients considering them as high-risk patients for developing hemorrhagic complications (grade B, EL 3).

#### Cancer

Several studies have suggested that there is a role of blood coagulation proteins in tumor progression. Hypercoagulable state is commonly seen in cancer patients is an important risk factor for thrombosis, and may also play a role in tumor progression and metastasis [178, 179]. The risk of thrombosis among cancer patients is about four times higher than in the general population and the risk increases to about 6.7-fold in patients receiving chemotherapy [180]. A number of anticancer agents, as well as of drugs used in supportive cancer therapy have been associated with an increased risk of VTEs. The combination of hormonal and chemotherapy seems to play a synergistic role in the development of thrombosis in patients with cancer [181]. Cancer patients with VTE are more likely to develop recurrent thromboembolic complications and major bleeding during anticoagulant treatment than those without malignancy. These risks correlate with the extent of the cancer [182, 183]. Several studies have shown the better feasibility of LMWH compared to warfarin in cancer patients in order to circumvent the problem of coumadin and chemotherapy interaction [184, 185]. Similar results were observed in a case report, wherein warfarin was replaced by LMWH [186]. Cancer patients who are on active anti-cancer therapy are at increased risk of bleeding due to chemotherapy-induced thrombocytopenia. Such interactions have been demonstrated in few reports [187–189].

### Indian recommendation

- In patients with chemotherapy, VKA therapy should be closely monitored (grade A, EL 2)

#### Renal impairment

As VKAs are metabolized in the liver, no dosage adjustments are required in patients with chronic renal impairment. However, a careful monitoring of therapy is recommended.

In patients with renal insufficiency, dosing and monitoring of anticoagulants should be considered carefully. Anti-Xa activity is also affected during renal impairment; it is prolonged in severe renal impairment (creatinine clearance < 30 mL/min) and, to a lesser extent, in moderate renal dysfunction (30–50 mL/min) [190]. Moreover, the risk of thrombotic and bleeding complications and prevalence of AF is high in patients with renal failure [191, 192]. Use of VKAs is shown to reduce ischemic complications without significant bleeding risk in patients with mild-to-moderate chronic kidney disease (CKD) and AF (195). Heparin derivatives depend largely on renal excretion; therefore, in patients with renal disorders, heparin dosage adjustment is necessary in order to avoid accumulation and hence over-anticoagulation [191].

Dosage adjustment for factor Xa inhibitors in patients with renal insufficiency is recommended by ACCP [122].

Indian recommendations

- Renal impairment patients should be closely monitored (grade B, EL 3).

## Management of prosthetic valve complications

### Thromboembolic events

Even with appropriate antithrombotic therapy, the reported annual risk of thromboembolic events in patients with a mechanical heart valve is 1–2% and with the bioprosthetic valve is 0.7% [193].

Transthoracic echocardiography (TTE) and transesophageal echocardiography (TEE) are important tests in the diagnosis of suspected prosthetic valve thromboembolism [194]. TTE is the first line examination to evaluate severe obstructive thrombosis, whereas TEE is the method of choice for the diagnosis of small prosthetic thrombosis [194]. The prosthetic valve should be considered the source of thromboembolism even if echocardiographic findings are unchanged in suspected cases [194]. Annual follow-up in patients with prosthetic heart valves should include a review of the adequacy of anticoagulation and any issues related to compliance with medical therapy. Screening questions for symptoms that may be related to embolic events are especially important if anticoagulation has been suboptimal [195]. Studies show that patients on VKA anticoagulation who are managed by a dedicated pharmacist-led anticoagulation clinic have lower rates of bleeding and thromboembolism compared with conventional monitoring by a clinician’s office [196, 197].

A cohort of patients (*N* = 25, 656) with aortic valve bioprostheses were evaluated for the risks and benefits of short-term anticoagulation (aspirin-only, 49%; warfarin-only, 12%; warfarin plus aspirin, 23%) by the Society of Thoracic Surgeons-Adult Cardiac Surgery Database (STS-ACSD). Patients treated with warfarin plus aspirin had a lower adjusted risk of death (relative risk (RR) 0.80, 95% confidence interval (CI), 0.66 to 0.96) and embolic event (RR 0.52, 95% CI, 0.35 to 0.76) but a higher risk of bleeding (RR 2.80, 95% CI, 2.18 to 3.60) relative to aspirin-only. Further, given the clear trade-off between thromboembolic and bleeding events, the study recommended the use of warfarin plus aspirin for bioprosthetic AVR patients [60]. Similarly, Mistiaen W et al. in their retrospective study also advocated aspirin in bioprosthetic AVR patients with larger valve size for prevention of thromboembolism. The long-term use of warfarin after bioprosthetic AVR could be cautiously considered in elderly patients with AF and previous thromboembolic events [198].

Measures to improve patient compliance, including patient education and more frequent monitoring, should be instituted. Patients should be educated about symptoms related to embolic events and instructed to promptly report to a healthcare provider as soon as the symptoms occur [175, 199–201].

European Association of Echocardiography (EAE) and ACC recommend TTE for diagnosis of thromboembolic events [10, 51, 202]. Thromboembolic events are managed by increasing the target INR range and adding additional antithrombotic drug. It also includes increasing patient compliance to anticoagulation therapy and thereby maintaining the adequate anticoagulation.

### Indian recommendations

- TTE is recommended for the diagnosis of thromboembolic events (grade A, EL 2).
- Treatment with tissue plasminogen activator (tPA) and heparin is recommended in patients with stroke; other vascular occlusions should be managed by surgery (grade C, EL 4).
- In anticoagulant patients with thromboembolic events, daily aspirin (75–81 mg) is recommended with an increase in the target INR range (mechanical AVR: 2.5–3, mechanical MVR: 3–4) (grade C, EL 4).
- In patients with bioprosthetic valve, who are only on aspirin, the addition of VKAs can be considered (grade B, EL 3).
- Measures to increase patient compliance (patient education) are recommended in all patients with thromboembolic events (grade C, EL 4).

### Thrombosis of prosthetic valves

The prevalence of mechanical valve thrombosis is 0.3–1.3% per pt-yr in developed countries and 6.1% per pt-yr in developing countries [203–205]. Bioprosthetic valve thrombosis is less common. The adsorption of plasma proteins, including fibrinogen, onto the surface of the valve prosthesis, leads to platelet adhesion and induction of the coagulation cascade [206]. Another etiological factor is a turbulent trans-prosthetic blood flow, an un-physiological flow that increases the blood-borne shear stress, which causes metabolic and structural damage to the endocardium making it susceptible to further thrombosis [206]. Similarly, blood stasis in re-circulation areas downstream of the prosthesis also favors thrombosis. Other device-free causes are clot-promoting factors including inadequate coagulation, AF, incomplete endothelialization of the valve sewing ring, and inflammation often with a raised fibrinogen level. Type of valve and location also affect the risk of thrombosis [206]. Thrombosis is 20 times more likely in the tricuspid position; mitral prosthetic valve thrombosis is 2–3 times more common than aortic prosthetic thrombosis. However, the introduction of the bileaflet mechanical valve and use of pyrolytic carbon as a valve material have reduced the risk of thrombosis as pyrolytic carbon is more biocompatible and thrombo-resistant and wear resistant [207].

Differentiation of valve dysfunction due to thrombus versus fibrous tissue growth (pannus) is challenging because of similar clinical presentations. TTE allows evaluation of valve hemodynamic and detection of valve stenosis or regurgitation. Leaflet motion and thrombus may be visualized in some patients. TTE also allows measurement of LV size and systolic function, left atrial (LA) size, right heart function, and an estimation of pulmonary pressures [205, 208]. A study by Habib G et al. comparing the efficacy of TTE and TEE in 23 prosthetic heart valve patients concluded that TTE was the procedure of choice in patients with severe obstructive prosthetic thrombosis, while the TEE approach appeared promising in partial thrombosis with mild or absent obstruction [209]. Thrombi on the LA side of the mitral valve can be imaged directly with TEE, which is obscured by shadowing in TTE imaging. Thrombi tend to be larger, less dense, and more mobile on ultrasound imaging than chronic fibrous ingrowth or pannus. Thrombus size measured with TEE is an independent predictor of outcome after thrombolysis of prosthetic heart valve obstruction [205, 210]. Irrespective of New York Heart Association (NYHA) class, patients with thrombus area < 0.8 cm^2^ are at lower risk for complications from thrombolysis. TEE should be used to identify lower-risk patients for thrombolysis [210]. ACC recommends TTE in patients with suspected prosthetic valve thrombosis to assess hemodynamic severity and resolution of valve dysfunction, and if the thrombus is detected, TEE can be used to assess thrombus size and valve motion.

The left-sided prosthetic heart valve thrombosis can be treated either by fibrinolytics or surgical intervention [205]. When treating left-sided PVT, the risks associated with reoperative surgery must be weighed against the risks of embolic complications and bleeding associated with the use of fibrinolytic therapy. In patients with recent hemorrhagic stroke, surgery is a better choice because of the bleeding risks associated with fibrinolysis. In patients with large, mobile clots that extend beyond the prosthesis, surgical intervention is better suitable than fibrinolysis, which is associated with significant risk of systemic embolism. Although RCTs have not been performed, the weight of the evidence favors surgical intervention for left-sided prosthetic valve thrombosis unless the patient is asymptomatic and the thrombus burden is small. Nevertheless, several studies also suggested heparin could be an initial option of treatment for the non-obstructive PVT [211, 212].

The fibrinolytic therapy of a left-sided obstructed prosthetic heart valve is associated with an overall rate 15–17% of bleeding risk [210, 213, 214]. Factors including active internal bleeding, history of hemorrhagic stroke, recent cranial trauma or neoplasm, diabetic hemorrhagic retinopathy, large thrombi, mobile thrombi, systemic hypertension (> 200 mmHg/120 mmHg), hypotension or shock, and NYHA class III to IV symptoms identify patients at risk for adverse outcomes of fibrinolytic therapy. The degree of risk is directly related to thrombus size. Thrombus area (2D TEE) > 0.8 cm^2^ and thrombus diameter 1.0 cm is associated with increased embolic risk and the rate of complications increases 2.4 fold per 1.0cm^2^ increase in size, which makes surgery better option [210]. The mortality rate in thrombosed prosthetic heart valve patients is reduced with prompt surgical treatment. Deviri E et al. in a retrospective study reported that perioperative mortality increased directly with NYHA class; 17.5% in NYHA IV and 4.7% in NYHA I to III. Mortality was similar for removing the thrombus or replacing the entire prosthetic valve [215]. In 2009, Roudaut A et al. presented one of the first studies that directly compared fibrinolysis to surgery in a large single-center retrospective study. These data suggest no difference between the approaches with respect to mortality, a much higher rate of embolic episodes in the fibrinolysis group, and less hemodynamic success. Furthermore, long-term freedom from recurrence was better in the surgical group [214]. Likewise, fibrinolysis was as successful as surgical intervention in normalizing hemodynamics in a non-randomized, retrospective cohorts of thrombosed mechanical, or biological tricuspid valve prostheses. Small pulmonary emboli appear to be well tolerated with fibrinolysis of right-sided valve thrombosis, and systemic emboli are uncommon [216, 217].

Clinical symptoms can be effectively ameliorated and normal hemodynamics can be restored with the prompt surgical treatment of a thrombosed prosthetic heart valve in patients who do not have a contraindication to surgical intervention. A meta-analysis of 7 studies with 690 cumulative episodes of left-sided prosthetic valve thrombosis showed that fibrinolytic therapy was able to restore normal valve function of only about 70% in 244 cases, whereas with surgery restoration rate was 86.5% in 446 cases. Statistically significant differences in the mortality rates were not observed between surgical and fibrinolytic therapy, but surgery was associated with lower rates of thromboembolism (1.6% vs. 16%), major bleeding events (1.4% vs. 5%), and recurrent prosthetic valve thrombosis (7.1% vs. 25.4%) [218].

In a retrospective study by Singh AK et al., thrombolytic therapy for the thrombosed prosthetic bileaflet mechanical valve at the mitral and the aortic position was compared [219]. Overall, 53% and 27% patients had complete and partial resolution with thrombolytic therapy and in 20% patients, no change was observed. The proportion of patients to full response to the fibrinolytic therapy was similar at both mitral and aortic position. Whereas, partial response was high in mitral position (31%) compared to aortic valve (17%). Thrombolysis avoided surgery in NYHA III/IV patients with almost 50% complete response. Management of PVT with thrombolytic therapy has also been demonstrated in various case reports and case series. Srinivas B et al. demonstrated successful management of acute prosthetic valve thrombosis post mitral valve replacement in the first trimester of pregnancy with IV streptokinase [220]. Likewise, Sharma V et al. reports the management of left-side PVT and Kuchulakanti PK et al. reports successful treatment of aortic prosthetic valve thrombosis, with tenecteplase [221, 222].

ACC and ACCP recommend fibrinolytics or surgical intervention for the management of PVT; the clinical judgment should be based on the position of the valve (left or right) and thrombus burden as assessed by echocardiography [38, 51]. The strategies for addressing PVT and recommended dose of fibrinolytics are represented in Table 10 [30, 51]. However, ACCP does not elaborate upon the doses of fibrinolytic therapy used. If fibrinolytic therapy is successful, ACC suggests following IV UFH until VKA therapy achieves an INR of 3.0 to 4.0 for aortic prosthetic valves and 3.5 to 4.5 for mitral prosthetic valves; with a structured institutional protocol with indications, contraindications, and a specific timeline for medication administration and patient monitoring. ACC suggest to change anticoagulation regimen as outlined in the management of thromboembolic events after assessment of the adequacy of anticoagulation and follow up of the same. CSI recommend a change from mechanical to bioprosthesis in patients having protein C or S deficiency as they are unsuited for Coumadin therapy. CSI also suggest a trial of direct oral thrombin inhibitor before resorting to surgery [10].

**Table 10:** The management strategies for prosthetic valve thrombosis (ACCP, ACC)

PVT	ACC [51]	ACCP [30]
Diagnosis	• Based on TTE and TEE for assessment of thrombus burden	• Not mentioned
Clinical judgment	• Based on thrombus burden and position of valve (left/right)	• Based on thrombus burden and position of valve (left/right)
Emergency surgery	Left-sided PVT • with a mobile or large thrombus (> 0.8 cm^2^) • with NYHA class III to IV symptoms	Left-sided PVT • with a mobile or large thrombus (> 0.8 cm^2^)
Fibrinolytics	Left-sided PVT • Very small thrombus- UFH, if persists give fibrinolytics • Thrombus (≤ 0.8cm^2^): fibrinolytics in patients with recent onset (< 14 days) of NYHA class I to II symptoms (class II, level B)	Left-sided PVT • Very small- UFH, accompanied by serial Doppler echocardiography to document thrombus resolution or improvement over other alternatives if persists give fibrinolytics • Thrombus (≤ 0.8cm^2^): fibrinolytics over surgery • In left-sided PVT with > 0.8 cm^2^, in whom surgery is contraindicated • In absence of contraindications to fibrinolytics, given in right-sided PVT
Fibrinolytic dosage	• tPA: 10 mg/20 mg IV bolus followed by 90/10 mg infused IV over 2/3 h • Heparin and glycoprotein IIb/IIIa inhibitors are held, aspirin can be continued • Alternatively, streptokinase 500,000 IU in 20 min followed by 1,500,000 IU over 10 h • Urokinase is less effective than tissue plasminogen activator or streptokinase	Not mentioned

*ACC* American College of Cardiology, *ACCP* American College of Chest Physicians, *IU* international units, *IV* intravenous, *NYHA* New York Heart Association, *PVT* prosthetic valve thrombosis, *tPA* tissue plasminogen activator, *TEE* transesophageal echocardiography, *TTE* transthoracic echocardiogram, *UFH* unfractionated heparin

### Indian recommendation

- TTE is recommended in patients with suspected prosthetic valve thrombosis to assess hemodynamic severity and resolution of valve dysfunction (grade B, EL 3). Cinefluoroscopy can be considered as an additional tool for diagnosis of prosthetic valve thrombosis (grade C, EL 4).
- TEE is recommended to assess thrombus size and valve motion (grade B, EL 3).
- UFH is recommended in a very small and non-obstructive thrombus burden of left-sided PVT if not treated fibrinolytics are recommended (grade B, EL 3).
- In patients with left-sided PVT, with thrombus burden ≤ 0.8 cm^2^ fibrinolytics are recommended over surgery (grade B, EL 3).
- Emergency surgery is recommended in case of left-sided PVT with a mobile or large thrombus > 0.8 cm^2^. Fibrinolytics can be considered in patients with contraindications to surgery (grade B, EL 3).
- The right-sided thrombosis can be treated with fibrinolytics if no contraindications to fibrinolytics are present. If fibrinolytic therapy is successful, IV UFH is recommended until the patient achieves an INR of 3.0 to 4.0 for aortic prosthetic and 3.5 to 4.5 for mitral prosthetic valves (grade B, EL 3).

### Bleeding complications

The incidence of major bleeding complications in patients with a mechanical valve and taking OAT varies from 0.34 to 1.32% per pt-yr [223]. The prominent features associated with anticoagulant overdose include bleeding (which may be manifested as nasal bleeds), hematemesis, hemoptysis, gastrointestinal bleeding, vaginal bleeding, hematuria, cutaneous hemorrhages, gingival bleeding, hematomata, and bleeding into joints or menorrhagia.

Numerous risk scores have been developed to help predict bleeding events considering other comorbidities such as previous gastrointestinal bleeding, CKD, previous stroke or MI, and anemia (Table 11). The majority of these risk scores have been developed to assess bleeding risk in patients with AF. The outpatient bleeding risk index has been prospectively validated (majority of the patients were prosthetic valve patients in validation study) and is often a useful tool for helping to guide clinician decision-making. Age is by far the strongest predictor of hemorrhage. The risk of bleeding appears to steadily increase once a patient is > 75 years old [228]. Pharmacogenetics also seems to influence the risk of bleeding events.

**Table 11:** Bleeding risk prediction scores

Risk score [source]	Bleeding risk factors	Risk classification points	Annual rate of major bleeding
Outpatient bleeding risk index [224]	Age ≥ 65 years, history of stroke, history of gastrointestinal bleeding, recent MI, or Hct < 30% or SCr > 1.5 mg/dl or diabetes 1 point each	Low: 0 Intermediate: 1–2 High: ≥ 3	Low: 3 Intermediate: 12 High: 30
Contemporary bleeding risk model [225]	(0.49 × age ≥ 70 years) + (0.32 × female sex) + (0.58 × remote bleed) + (0.62 × recent bleed) + (0.71 × alcohol/drug abuse) + (0.27 × diabetes) + (0.82 × anemia) + (0.32 × antiplatelet therapy)*	Low: ≤ 1.07 Intermediate: 1.07–2.18 High: > 2.19	Low: 0.9 Intermediate: 2.0 High: 5.4
Has-bled [226]	Hypertension (SBP > 160 mmHg), abnormal renal/liver function, stroke, history of bleeding, labile INR, elderly age (> 65 years), drugs (antiplatelet/NSAIDS/alcohol) 1 point each	Low: 0–1 Intermediate: 2 High: ≥ 3	Low: 1.1 Intermediate: 1.9 High: 4.9
Hemorrhages [227]	Hepatic or renal disease, ethanol abuse, malignancy, older age (> 75 years) reduced platelet count/function, re-bleeding, uncontrolled hypertension, anemia, genetic factors (CYP2C9 SNP), excessive fall risk, stroke 1 point each	Low: 0–1 Intermediate: 2–3 High: ≥ 4	Low: 2.1 Intermediate: 5 High: 8.8

*NSAID* non-steroidal anti-inflammatory drugs, *MI* myocardial infarction, *SBP* systolic blood pressure

Excessive anticoagulation (INR ≥ 5) greatly increases the risk of hemorrhage. In patients with acute or prior intracerebral hemorrhage, antithrombotic therapies present a clinical dilemma with competing risks and benefits. In a retrospective study, Phan and associates found that discontinuation of anticoagulation therapy for 1–2 weeks had a low probability of thromboembolic events in patients with high embolic risk [229]. Similarly, another report suggests interruption of anticoagulation therapy as safe in patients with intracerebral hemorrhage and mechanical heart valves but without previous evidence of systemic embolization [230]. In major bleeding cases, the risk of bleeding and valve thrombosis must be weighed and the period of discontinuing anticoagulation should be adjusted based on individual case [231].

In a study on Indian patients with MVR, the incidences of bleeding complication are common in patients with a mechanical valve (6%) than those with a bioprosthetic valve (0.9%) [61]. Evidence suggests that fresh frozen plasma (FFP) and packed red cell transfusion are used as antithrombotic therapies for the management of bleeding incidences in prosthetic valves patients in India [41, 61, 64].

Several RCTs report the efficacy of vitamin K for the management of bleeding complications in patients with excessive anticoagulation therapy [232, 233]. Yiu KH et al. demonstrate low dose intravenous vitamin K as a safe alternative to FFP infusion for warfarin overdose in patients with mechanical heart valves [232]. Lubetsky A et al. show that oral administration of vitamin K1 is as effective and safe as intravenous administration in patients with excessive anticoagulation [233]. Moreover, the addition of oral vitamin K1 along with omitting warfarin significantly reduces the time to achieve INR of 4.0 in patients with excessive anticoagulation [234]. Overall, high dose vitamin K therapy (5–10 mg) and large volume type-specific FFP therapy are the effective treatment options for controlling the INR in mechanical heart valve patients with warfarin-induced major bleeding complications [223].

**Fig. 3:**
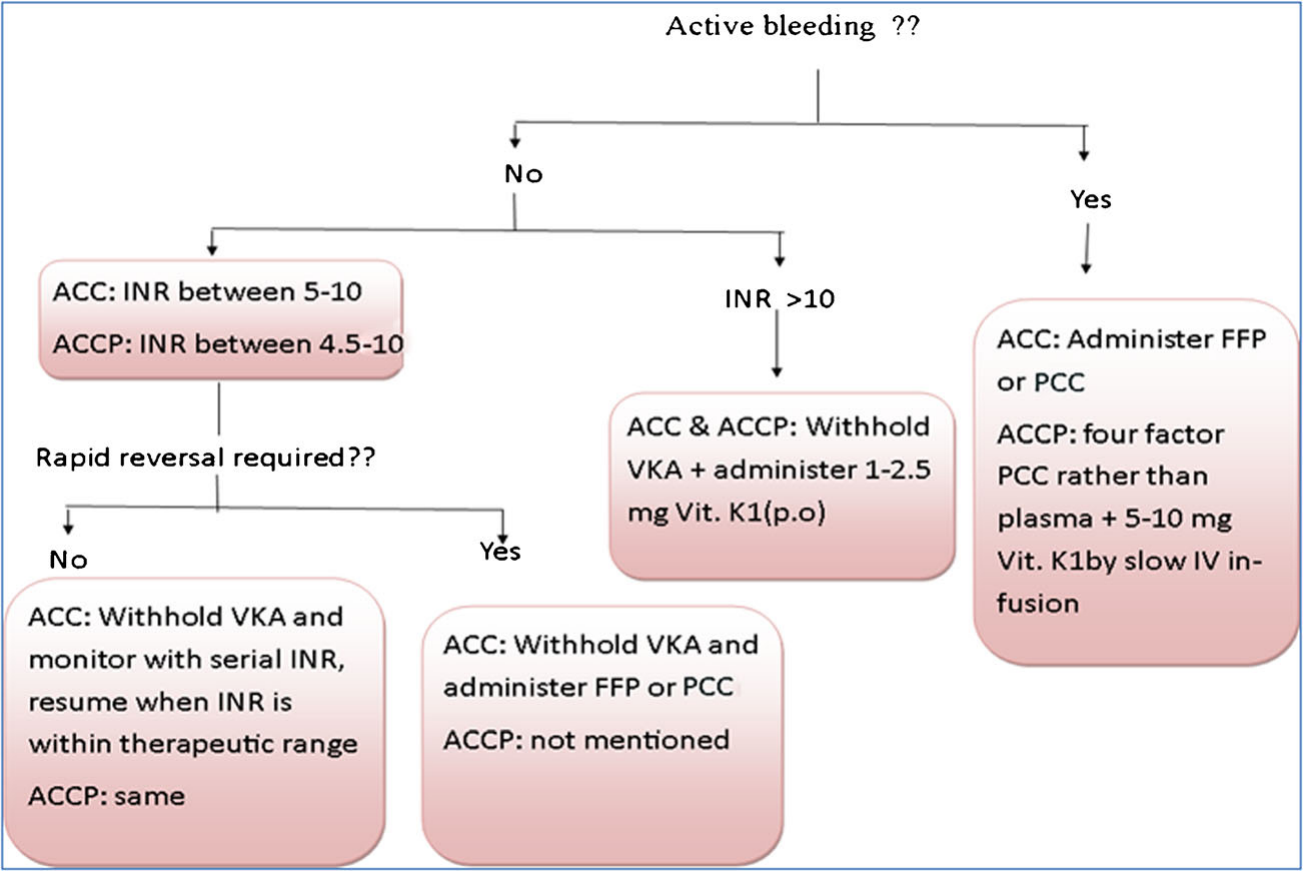
Management of bleeding events in prosthetic valve patients (ACC, ACCP)

Management of bleeding events as recommended by ACC and ACCP are presented in Fig. 3. For patients initiating VKA therapy, ACCP suggests against the routine use of clinical prediction rules for bleeding as the sole criterion to withhold VKA therapy. For patients with VKA associated major bleeding, ACCP suggests rapid reversal of anticoagulation with four-factor prothrombin complex concentrates (PCC) rather than with plasma [51, 88].

### Indian recommendation

- In absence of active bleeding and INR in the range of 4.5–10.0, it is recommended withholding VKA with serial INR determination, and resuming when INR is therapeutic (grade B, EL 3).
- In absence of active bleeding, and INR > 10, it is recommended withholding VKA and administering FFP and should be hospitalized (grade C, EL 4).
- For patients with VKA-associated active bleeding, withhold VKA, and administer FFP. It is also recommended to administer vitamin K1 as slow IV infusion if uncontrolled (grade A, EL 2).

### Prosthetic valve endocarditis

Prosthetic valve endocarditis (PVE) is a serious complication of cardiac valve replacement and is an important cause of morbidity and mortality. After excluding other common causes of fever, echocardiography should be performed in all cases where there is a medium or high clinical suspicion or when the patient is severely ill [235]. Durack and colleagues proposed a diagnostic criterion which combines echocardiographic findings with clinical and microbiological data [236]. Echocardiographic findings that were considered to be major criteria for the diagnosis of IE includes (1) presence of vegetations which are defined as irregularly shaped, independently mobile structures with relatively low echogenicity that are implanted in a valve or mural endocardium in the trajectory of a regurgitant jet or implanted in a prosthetic material with no alternative anatomical explanation; (2) presence of abscesses; or (3) presence of a new dehiscence of a valvar prosthesis [236]. Vegetations formed in the valve ring area may spread to the leaflet of the prosthetic valve, stent, or occluder, could impair the opening and closing of the valve. Without considering the echocardiographic findings in the context of the clinical presentation, differentiation of vegetations from other masses, such as thrombus, sutures, or pledgets, might be difficult [237]. Echo-lucent or an echo-dense mass produce abnormal cavities, which can be seen in the valve ring area. Abscesses may occasionally infiltrate the septum and impair the conduction system and are sometimes observed even in cases in which vegetations are absent. Edema and hematoma might occur early after surgery, particularly in stentless valves, and simulate a walled-off abscess. Progression of an abscess may result in a fistula between the heart chambers. Shunt detection in such cases can be facilitated by color Doppler [237]. Suture dehiscence and paravalvular regurgitation in all prosthetic valves and valve destruction in bioprosthetic valves can be caused due to IE [235].

TEE was demonstrated superior to TTE in assessing PVE and its complications. TEE has been shown to have high sensitivity (86–94%) and specificity (88–100%) for the detection of vegetations [238–241]. Secondary destruction of leaflet tissue may lead to infection of bioprosthetic valve leaflets [235]. It is often difficult to distinguish between wear-and-tear degeneration of tissue valves and IE. TEE helps in improving the diagnostic accuracy of IE on bioprosthetic valves [242]. TEE is also superior to TTE in detecting associated perivalvular abscesses in the posterior but not in the anterior aortic root (because of shadowing, depending on valve location) [243–245]. The reported sensitivity and specificity for the diagnosis of perivalvular abscesses with TTE are 28% and 98% respectively, and with TEE are 87% and 95%, respectively [243]. TEE is, therefore, necessary in cases in which IE is strongly suspected, even when no significant findings are seen on TTE [246]. Multiple TEE planes combined with TTE views must be exploited to minimize the risk of missing a significant finding when images are technically difficult to obtain. When both TEE and TTE studies are negative, there is a 95% negative predictive value [247, 248]. The thromboembolic risk was reported higher with PVE than that of native valve IE, i.e., rates between 50 and 88% [38, 249, 250]. Antimicrobial therapy remains the mainstay of therapy; however, most patients require surgical removal and replacement of infected prosthesis. Furthermore, the postponement in the prevention of embolization is allied with stroke within 3 days of diagnosis [250]. The risk of embolism is related to the size, and mobility of vegetation, especially patients with very mobile and large (> 15 mm) vegetations are at higher risk. Moreover, the risk of new embolism is highest during the first days of antibiotic therapy and decreases after 2 weeks [194]. Roudaut A et al. have demonstrated performing diagnostic blood cultures in the presence of fever to rule out IE [205]. In a case-control study, Agarwal S et al. identified the risk factors for PVE in Indian population. These risk factors were functional NYHA class III or IV, history of smoking, prior history of IE, fever in the intensive care unit (ICU), wound infection, and gastrointestinal bleeding [251]. There are no randomized studies assessing the impact of antithrombotic therapy on PVE. However, the results of observational studies suggest that the risk of continuing anticoagulation outweigh the benefits [250, 252–255]. Carpenter JL et al. compared the outcomes of anticoagulation in native valve IE (*N* = 82) with PVE patients (*N* = 18). The study reported that 36% of patients with PVE had symptomatic cerebral hemorrhage during anticoagulation continuation and 80% of them died. Furthermore, central nervous system (CNS) hemorrhage was the principal cause of death in patients with PVE [256].

Two different recommendation statements from ESC [194, 257] and one AHA statement [258] suggest performing TTE in suspected IE. In the case of negative TTE in suspected PVE, TEE is recommended. A repeated TTE/TEE within 7–10 days is recommended in patients with high suspicion of IE if initial examinations are negative. ACC and ESC recommend using Modified Duke criteria in evaluating a patient with suspected IE [51, 194]. ACCP recommended that “In patients on VKA for a prosthetic valve who develop IE, VKA can be discontinued at the time of initial presentation until it is clear that invasive procedures will not be required and the patient has stabilized without signs of CNS involvement. When the patient is deemed stable without contraindications or neurologic complications, reinstitution of VKA therapy is suggested” [38]. Prosthetic cardiac valve implantation has been indicated as one of the conditions associated with the highest probability of adverse outcomes from IE [259, 260]. According to the guideline, IE prophylaxis is not required in routine anesthetic injections through non-infected tissue, dental radiographs, prosthodontic or orthodontic appliances, adjustment of orthodontic appliances replacement or removable, orthodontic brackets placement, deciduous teeth shedding, and bleeding from trauma to the lips or oral mucosa [259]. Moreover, prophylaxis is also not recommended for bronchoscopy unless the respiratory tract mucosa is incised [259].

- Procedures requiring antibiotic prophylaxis [259, 260]:
  1. Dental procedures: all dental procedures that involve dental-gingival manipulation, and management of a periapical region of teeth or oral mucosa perforation.
  2. Respiratory tract procedures: incision or biopsy during tonsillectomy and adenoidectomy.
  3. Procedures for infected skin, skin structure, or musculoskeletal tissue

### Indian recommendations

#### Diagnosis

- TTE is recommended in suspected IE, in the case of negative TTE in suspected PVE, TEE is recommended. If initial examinations are negative, repetition of TTE/TEE is recommended within 7–10 days in patients with high suspicion of IE. Modified Duke criteria should be used in evaluating a patient with suspected IE (grade B, EL 3).

#### Prophylaxis

- Antibiotic prophylaxis is recommended for certain dental procedures like gingival or periapical (root) procedures with perforation of the mucosa, and also for infected GI and urogenital tract procedures (grade C, EL 4).

#### Antithrombotic

- It is recommended to discontinue VKA, in patients on VKA for a prosthetic valve who develop IE, until it is clear that invasive procedures will not be required and the patient has stabilized without signs of CNS involvement. When the patient is deemed stable without contraindications or neurologic complications, reinstitution of VKA therapy is recommended (grade B, EL 3).

## Follow-up evaluations and management of concomitant cardiac disease

Congestive heart failure (CHF), coronary arterial disease (CAD), and AF are the frequent complications encountered in patients with valve replacement. CAD and arrhythmias are the more commonly combated hitches in AS and mitral valve disease patients respectively [10]. Data regarding the optimal follow-up management after valve surgery is limited. Therefore, physician preference and experience are inevitable for the clinical practice [261]. Therefore, a consensus regarding special management of concomitant heart diseases with follow-up and evaluation of progressive diseases is highly desirable in the patients with VHD.

### Heart failure

Patients with heart valve prostheses have substantial cardiac compromise and are expected to be at noticeable risk for CHF [10]. Following valve surgery, CHF may be responsible for further complications like prosthetic-related complications, deterioration of repair, LV dysfunction, or progression of another valve disease. Therefore, it is likely to be very crucial to manage CHF during follow-up for VHD.

The ACC/AHA categorized CHF into four stages incorporating both risk factors and abnormalities of cardiac structure [51]. Stage A patients are at high risk for HF without any structural heart disease; however, stage B and C are the patients with any structural heart disease. Valve patients should be treated as stage C or D preoperatively and stage B or C following surgery. Similarly, the NYHA classifies CHF according to the severity of HF and provides the guidance for patient management. Stage B and C patients are generally managed with moderate doses of angiotensin-converting enzyme (ACE) inhibitors and β blockers along with antithrombotics [10]. However, in uncontrolled CHF cases, the addition of spironolactone and digoxin may be considered. Furthermore, in patients with severely decompensated AS with NYHA class IV HF symptoms, vasodilator therapy may be recommended [51].

### Indian recommendations

- Antithrombotics are recommended in valve replacement patients with stage A heart failure (grade A, EL 1). ACE inhibitors and β blockers could be beneficial if added in older patients (grade B, EL 3)
- ACE inhibitors and β blockers along with antithrombotics are recommended in patients with stage B, C heart failure (grade A, EL 1). Moreover, spironolactone and digoxin can be added if congestive failure supervenes despite regular medications (grade A, EL 2).
- In patients with stage D heart failure, other interventions including cardiac transplant are recommended (grade D, EL 4).

### CAD

Concomitant VHD and CAD are usually problematic in elder populations. The prevalence of mitral regurgitation (MR) and AS in patients aged ≥ 70 years is reported as 10% and 4% respectively. Furthermore, in AS patients, the prevalence of concurrent CAD has been > 50% and > 65% in patients with age > 70 and > 80 years respectively. In addition, elderly patients often have more comorbidity with previous cardiac operations; and the CABG during valve surgery could reciprocate and doubles the operative risk of the procedure [262].

In an era of drug-eluting stent (DES) usage, the concurrent use of dual APAs with VKAs has been a major issue in patients with percutaneous coronary intervention (PCI). The existing guidelines do not address these issues. Approximately 5% of patients requiring PCI also present with an indication for OAT. In such cases, the type of stent selected, the use of oral anticoagulants, APA, or their combinations, the target INR, and the duration of treatment are essential considerations in relation to the risk of thrombosis/thromboembolic events and bleeding risk. Whenever possible, triple therapies with shorter durations are preferred over longer durations [263]. Moreover, pertaining to shorter duration of triple antithrombotic therapy, a bare-metal stent (BMS) may offer the advantages of lower bleeding risk over DES in patients with non ST segment elevation acute coronary syndrome (NSTE-ACS) and necessitate OAT for AF, mechanical heart valve, deep venous thrombosis (DVT), or other conditions [264, 265]. However, these recommendations do not specify the optimal treatment according to the various indications for anticoagulation or the duration of treatment, nor do they take into account the estimated thrombosis and bleeding risks. On the other hand, a meta-analysis including patients with DES versus BMS with indications for OAT reveals that there were no significant differences between DES and BMS for a pooled estimate of hazard ratio (HR) for the major adverse cardiac event (MACE), all-cause mortality, MI, and bleeding complications [266].

The WOEST (What is the Optimal Antiplatelet and Anticoagulant Therapy in Patients with Oral Anticoagulation and Coronary Stenting) trial is the first published study to demonstrate the optimal APA therapy in patients taking OAT. Patients (*N* = 563, from this 25% are NSTE-ACS) randomized to single APA (clopidogrel) were associated with significantly less bleeding complications and no increase in thrombotic events than those randomized to dual APAs (aspirin and clopidogrel) [267]. The target INR should be lowered to 2.5 to 3.0 for mechanical heart valves and to 2.0 to 2.5 for other indications during triple therapy treatment for PCI [268]. Schematic algorithms have also been suggested for selection of an appropriate approach to decide on interventions in patients who are on anticoagulation and undergoing stenting [263, 267, 269].

### Indian recommendations

- Bare metal stents are recommended over drug-eluting stents in patients with valve replacement to lower bleeding risk (grade D, EL 4).

### Atrial fibrillation

The estimated prevalence of postoperative atrial fibrillation (POAF) is approximately 30% after CABG surgery, 40% after valve replacements or repair, and up to 50% after combined CABG and valvular procedures [270, 271]. In a large prospective trial, the Prophylactic Oral Amiodarone for the Prevention of Arrhythmias that Begin Early After Revascularization, Valve Replacement, or Repair (PAPABEAR), amiodarone (10 mg/kg daily) was compared with placebo. The study concluded that prophylaxis with oral amiodarone was effective and safe in all patients for prevention of AF [272]. Similarly, a meta-analysis of 19 trials comparing prophylactic amiodarone versus placebo for prevention of AF after cardiac surgery has demonstrated a significant reduction in AF (reduced by 50% in the amiodarone group), ventricular tachyarrhythmia, strokes, and hospital stay. The authors concluded that in the absence of a contraindication, prophylactic amiodarone should be implemented as a routine therapy for high-risk patients undergoing cardiac surgery. A total dose of 5 to 10 g was sufficient irrespective of the timing of initiation (preoperative, intraoperative, or postoperative) or route of administration (oral, IV, or both). Likewise, the scheduled duration of therapy was short in the postoperative period (< 2 weeks) [273]. Furthermore, the addition of β blockers may help in the postoperative period by reducing sympathetic triggers of AF [274]. Kar SK et al. evaluated the effect of prophylactic amiodarone in patients with RHD undergoing valve replacement surgery. The study reported that a single dose (3 mg/kg) of intraoperative amiodarone may be used to decrease postoperative arrhythmia in patients with valve replacement surgery [275]. Furthermore, a single intraoperative dose (3 mg/kg) of IV amiodarone has improved the rate of conversion from AF to normal sinus rhythm, decreased both the need and energy requirement for cardioversion/defibrillation, and decreased the AF recurrence within 1 day [276].

The European Association for Cardio-thoracic Surgery (EACTS) recommends the perioperative use of β blockers as the first choice in all patients undergoing cardiac surgery, unless otherwise contraindicated and amiodarone in whom β-blocker therapy is not possible [277].

### Indian recommendations

- Prophylactic amiodarone is recommended as a routine therapy for high-risk patients undergoing cardiac surgery in absence of a contraindication (grade A, EL 1).
- Amiodarone at a dose of 100–200 mg daily for 3 months with dosage monitoring along with β blockers is recommended for transient perioperative AF (grade A, EL 2).

### Follow-up cardiac evaluation

Doppler echocardiography at baseline (before discharge) serves as a reference for subsequent examinations in patients with valve replacement. The measurements should include regional and global left and right ventricular systolic function and size, diastolic LV function (not assessable with mitral valve prosthesis), atrial size, function of the native valves, pulmonary artery pressure estimates, pressure gradient across the newly implanted prosthesis, effective valvar orifice area, and presence of paravalvular leaks [278]. The first postoperative workup should include patient’s medical history, physical examination, electrocardiography (ECG), echocardiography, chest x-ray, complete blood count (CBC), creatinine, electrolytes, lactate dehydrogenase, and INR [278, 279]. The follow-up schedule generally depends upon the patient’s progress and accessibility to local facilities. However, all patients should continue to be followed-up at a cardiac center to identify prosthetic function deterioration, regurgitation recurrence, or disease progression at another valve site, any of which can occur with or without any change in symptoms [279]. Following patients require echocardiography at clinic visit [279]:

- Previously recognized abnormality that necessitates monitoring for progression/response to treatment, e.g., MR, sewing ring thrombus, previous IE, etc.
- New symptoms that advocate prosthetic dysfunction, progression of another valve lesion, or worsening LV function. Along with TTE, TEE and cinefluoroscopy may also be used in suspicion of prosthetic dysfunction. In bileaflet and tilting disc valves, cinefluoroscopy is used to identify the early limitation of leaflet movement.
- Bioprostheses, homografts, or autografts, to identify structural deterioration.
- In case of the Ross procedure, to identify aortic root dilatation, progressive aortic regurgitation (AR), or structural deterioration in the pulmonary homograft.
- In case of Marfan’s syndrome, to identify progressive dilatation of the aorta or progressive MR.

ESC recommends “first postoperative visit to the hospital or a cardiac specialist within 6 weeks of discharge if there has been no period of inpatient rehabilitation or within 12 weeks if a rehabilitation programme has been completed” [279]. The ACC recommends annual follow-up for cardiac history and physical examination in an asymptomatic uncomplicated patient [51]. Furthermore, an echocardiographic examination should be performed within 6 weeks to 3 months after valve implantation [51], while American Society of Echocardiography (AS-Echo) guideline recommends a baseline TTE at discharge or 2–4 weeks after hospital discharge, as an essential component of the first postoperative visit. Both guidelines recommend further follow-up by TTE and/or TEE if clinical symptoms or signs suggestive of prosthetic valve dysfunction or other cardiac pathology persist; with preference to TTE for initial examination [51, 202]. ACC advocates that there should not be any echocardiographic testing after the initial postoperative evaluation in stable mechanical valve patients, who do not have any symptoms or clinical evidence of prosthetic valve or ventricular dysfunction or dysfunction of other heart valves [51]. However, the frequency of echocardiographic testing has not been indicated by ACC; however, AS-Echo recommends annual follow-up in mechanical valve patients in the presence of a change in clinical status [202]. ACC recommends annual TTE after the first 10 years, while AS-Echo recommends annual echocardiography after the first 5 years in bioprosthetic valve patients, even in the absence of a change in clinical status [51, 202].

### Indian recommendations

- A first postoperative visit to a cardiac specialist should be within 1 month of discharge (grade A, evidence level 4).
- The timings of echocardiographic examination: first, at pre-discharge; second, at 1 month; then yearly; and should be done at any time when symptoms occur (grade C, EL 4).
- The patient should be followed up by TTE and/or TEE in case of clinical symptoms or signs of prosthetic valve dysfunction (grade C, EL 4).
- Echocardiographic testing is recommended for (a) unexplained cardiac symptoms, and (b) annually in all patients with CHF. Moreover, echocardiography is indicated whenever there is an episode of thromboembolism (grade C, EL 4).
- Mechanical heart valve patients should undergo annual follow up in presence of a change in clinical status (grade C, EL 4).

### CT and MRI scan-post valve implantation

Metals, polymers, and carbons are used in the preparation of prosthetic heart valves. Numerous studies have evaluated the safety of magnetic resonance imaging (MRI) examinations with a scanner that operates at ≤ 1.5Tesla in heart valve prostheses [280–282]. They found relatively minor magnetic field interactions in relation to the static magnetic fields of the MRI systems. The forces put forth by a magnetic field on these valves have been found to be less than the forces exerted by gravity. These forces were substantially lower than those exerted by the beating heart and resultant pulsatile blood flow [283]. Consequently, these patients are unlikely to be at risk for valve dehiscence. There is a hypothetical probability of electromagnetic interaction with metal in valves that may cause interruption of opening and closing of valves (referred to as the Lenz effect). However, no such cases have been reported in clinical practice with the low magnetic field [284]. Moreover, heating due to MR system was reported to be minor [280–282, 285]. Furthermore, image interpretation is rarely influenced by the artifacts provoked by mechanical valves [283]. In a cohort study, the safety of MR imaging was evaluated in patients who had undergone prior cardiac surgery. Out of 25 mechanical valve patients included, none of them developed adverse cardiac events, such as arrhythmia or worsening cardiac function, related to MRI [249]. Consequently, prosthetic heart valves, as well as metal sternal sutures and mediastinal clips, should not be considered as contraindications for an MRI ≤ 3 T any time after implantation [286, 287].

According to AHA scientific statement, the presence of a prosthetic heart valve or annuloplasty ring that has been formerly evaluated for MRI safety should not be considered as a contraindication to an MRI ≤ 3 T (and possibly even 4.7 T in some cases) any time after implantation [287]. MRI examination of patients with sternal wires is generally considered to be safe. Patients with IE and risk of valve dehiscence cannot undergo MRI [10].

### Indian recommendations

- MRI examination (3 T or less) is safe in patients with a prosthetic heart valve or annuloplasty ring or sternal wire (grade C, EL 3).
- MR examination for patients with risk of endocarditis and valve dehiscence should be decided in consultation with a radiologist (grade D, EL 4).

## Abbreviations

AACE: American Association of Clinical Endocrinologists
AATS: American Association for Thoracic Surgery
ACC: American College of Cardiology
ACCF: American College of Cardiology Foundation
ACCP: American College of Chest Physicians
AE: Adverse events
AF: Atrial fibrillation
AHA: American Heart Association
APA: Antiplatelet agent
AR: Aortic regurgitation
AS: Aortic stenosis
AS-Echo: American Society of Echocardiography
AVR: Aortic valve replacement
BMS: Bare-metal stent
CABG: Coronary artery bypass graft
CAD: Coronary arterial disease
CHF: Congestive heart failure
CHV: Chitra prosthetic heart valve
CKD: Chronic kidney disease
CNS: Central nervous system
CSI: Cardiological Society of India
DES: Drug-eluting stent
DVR: Dual valve replacement
DVT: Deep venous thrombosis
EACTS: European Association for Cardio-Thoracic Surgery
EAE: European Association of Echocardiography
EHRA: European Heart Rhythm Association
ESC: European Society of Cardiology
FFP: Fresh frozen plasma
GCPR: Good clinical practice recommendations
HF: Heart failure
IACTS: Indian Association of Cardiovascular-Thoracic Surgeons
ICD: Implantable cardioverter–defibrillator
IE: Infective endocarditis
INR: International normalized ratio
JAPI: Journal of The Association of Physicians of India
LMWH: Low molecular weight heparin
LV: Left ventricular
MACE: Major adverse cardiac event
MAV: Mechanical aortic valve
MI: Myocardial infarction
MR: Mitral regurgitation
MRI: Magnetic resonance imaging
NSAIDS: Nonsteroidal anti-inflammatory drugs
NYHA: New York Heart Association
OAT: Oral anticoagulation therapy
PCC: Prothrombin complex concentrates
PCI: Percutaneous coronary intervention
POAF: Postoperative atrial fibrillation
POC: Point-of-care
PSM: Patient self-management
PT: Prothrombin time
PVE: Prosthetic valve endocarditis
PVI: Pulmonary vein isolation
QOL: Quality of life
RCT: Randomized controlled trials
RHD: Rheumatic heart disease
SCAI: Society for Cardiovascular Angiography and Interventions
STS: Society of Thoracic Surgeons
TAVI: Transcatheter aortic valve implantation
TEE: Transesophageal echocardiography
TIA: Transient ischemic attack
TPA: Tissue plasminogen activator
TR: Therapeutic range
TTE: Transthoracic echocardiography
TTR: Target therapeutic range
TVR: Tricuspid valve replacement
UFH: Unfractionated heparin
VHD: Valvular heart disease
VKA: Vitamin K antagonist
VTE: Venous thromboembolism
